# Effect of Temperature Downshift on the Transcriptomic Responses of Chinese Hamster Ovary Cells Using Recombinant Human Tissue Plasminogen Activator Production Culture

**DOI:** 10.1371/journal.pone.0151529

**Published:** 2016-03-18

**Authors:** Andrea Bedoya-López, Karel Estrada, Alejandro Sanchez-Flores, Octavio T. Ramírez, Claudia Altamirano, Lorenzo Segovia, Juan Miranda-Ríos, Mauricio A. Trujillo-Roldán, Norma A. Valdez-Cruz

**Affiliations:** 1 Departamento de Biología Molecular y Biotecnología, Instituto de Investigaciones Biomédicas, Universidad Nacional Autónoma de México, Ciudad de México, México; 2 Unidad Universitaria de Apoyo Bioinformático, Instituto de Biotecnología, Universidad Nacional Autónoma de México, Cuernavaca, Mor. México; 3 Departamento de Medicina Molecular y Bioprocesos, Instituto de Biotecnología, Universidad Nacional Autónoma de México, Cuernavaca, Mor. México; 4 Escuela de Ingeniería Bioquímica, Pontificia Universidad Católica de Valparaíso, Valparaíso, Chile; 5 Departamento de Ingeniería Celular y Biocatálisis. Instituto de Biotecnología, Universidad Nacional Autónoma de México, Cuernavaca, Mor. México; Glasgow University, UNITED KINGDOM

## Abstract

Recombinant proteins are widely used as biopharmaceuticals, but their production by mammalian cell culture is expensive. Hence, improvement of bioprocess productivity is greatly needed. A temperature downshift (TDS) from 37°C to 28–34°C is an effective strategy to expand the productive life period of cells and increase their productivity (*q*_p_). Here, TDS in Chinese hamster ovary (CHO) cell cultures, initially grown at 37°C and switched to 30°C during the exponential growth phase, resulted in a 1.6-fold increase in the *q*_p_ of recombinant human tissue plasminogen activator (rh-tPA). The transcriptomic response using next-generation sequencing (NGS) was assessed to characterize the cellular behavior associated with TDS. A total of 416 (q > 0.8) and 3,472 (q > 0.9) differentially expressed transcripts, with more than a 1.6-fold change at 24 and 48 h post TDS, respectively, were observed in cultures with TDS compared to those at constant 37°C. In agreement with the extended cell survival resulting from TDS, transcripts related to cell growth arrest that controlled cell proliferation without the activation of the DNA damage response, were differentially expressed. Most upregulated genes were related to energy metabolism in mitochondria, mitochondrial biogenesis, central metabolism, and avoidance of apoptotic cell death. The gene coding for rh-tPA was not differentially expressed, but fluctuations were detected in the transcripts encoding proteins involved in the secretory machinery, particularly in glycosylation. Through NGS the dynamic processes caused by TDS were assessed in this biological system.

## Introduction

Recombinant proteins that require posttranslational modifications, similar to those found in humans, are preferentially produced in mammalian cells [1]. Chinese hamster ovary (CHO) cells have been used as the preferred heterologous expression system to produce recombinant glycoproteins, which are in increasingly high demand for the treatment of various diseases [1,2]. Hence, there exists a critical need to improve these bioprocesses in order to increase recombinant protein yields while maintaining product stability, activity, and biosafety [1,3,4]. One strategy used to improve specific and volumetric productivity is to decrease the culture temperature [5–8]. Effects of temperature downshifts (TDS) from 37°C to temperatures ranging from 28°C to 34°C (a procedure known as “biphasic cultures under moderate hypothermia”) have been reported, with the optimal conditions dependent on the product and cell line under investigation [9,10]. TDS usually reduces cell growth but increases cell viability, slows cell metabolism, delays cell death, and prolongs RNA half-life. Such effects have been related to increases in recombinant protein production [2,3,10–13].

Recent transcriptomic and proteomic studies have increased our understanding of the genetic and physiological processes in CHO cell cultures [14,15]. A transcriptomic analysis of a moderate hypothermic effect in CHO cells was performed using mouse and rat DNA microarrays [16–18]. The expression of 301 genes (from 1,655 known genes in rat cDNA chips) and 162 genes (from 4,643 known genes in mouse cDNA chips) were modified in CHO cells that were cultured at 33°C, as compared to those cultured at 37°C [16]. Other recombinant CHO cells under TDS showed 237 genes that were differentially expressed from 4,509 well-annotated genes using CHO cDNA microarrays [18].

Next-generation sequencing (NGS) technologies are more effective for deep sequencing analyses of genomes and transcriptomes compared to Sanger-based sequencing techniques, and are not limited by DNA chips [19,20], allowing the identification of a large number of RNA transcripts. For the CHO-K1 cell line genome, Illumina´s sequencing platform showed an estimate size of 2.45-Gb and 24,383 predicted genes [21,22], allowing a better description of the transcriptome data set [19,23]. Recently, data were obtained using NGS on CHO cultures undergoing TDS from 36°C to 31°C, which caused a decrease in protein productivity [24].

Since high productivity is associated with particular cell responses and different transcriptomic states [18,25], we aimed to analyze and illustrate the effects of TDS on the transcriptomic profile of CHO TF70R cells. TDS performed in this study resulted in an increased production of recombinant rh-tPA and viability, as also observed by others using the same cell line [10,26,27]. The effects of TDS on cell metabolism and the secretion pathway through the endoplasmic reticulum (ER) were also determined and described here. The NGS analysis shown is helpful for the development of clones with high productivity. In particular, it is proposed that specific changes in the ER and Golgi are the main responses associated to the increased recombinant protein production observed in the biological system used here.

## Materials and Methods

### Cell lines and culture conditions

The cell line, CHO TF70R, which produces rh-tPA, was obtained from Pharmacia & Upjohn S.A. Sweden and was a kind gift of Torsten Björlig. The cells were cultured in serum-free, chemically defined CD-OptiCHO^®^ medium (GIBCO ThermoFisher, Carlsbad, CA, USA), supplemented with 6 mM glutamine (Biowest LLC, Kansas City, MO, USA), 4 mg/mL insulin HUMULIN (Eli Lilly, Indianapolis, IN, USA), 100 U/mL penicillin (Antibióticos de México, México D.F., México), 0.01 mg/mL streptomycin (Sigma-Aldrich, Hamburg, Germany), 0.001 g/L Pluronic^®^ F-68 (Sigma-Aldrich, Hamburg, Germany), 200 nM methotrexate (Pfizer, New York, NY, USA), and 4% yeast extract (Becton Dickinson, Franklin Lakes, NJ, USA) at pH 7.0. Inocula were prepared in 75-cm^2^ T-flasks (Nunc, Roskilde, Denmark) and incubated at 37°C with humidified air containing 5% CO_2_. Six spinner flasks were inoculated with 3.5 × 10^5^ cells/mL at 90% viability in a working volume of 100 mL, and were cultured at 90 rpm with 5% CO_2_ atmosphere in a humidified incubator. After 48 h, three spinners were switched to 30°C (biphasic cultures) and three were kept at 37°C (control cultures). Samples were taken every 24 h until viability dropped to 40%. The cell concentration and cell viability were determined using a cell counter (TC10 BioRad, Hercules, CA, USA) and a Neubauer chamber following the trypan blue dye exclusion method.

### Determination of glucose, glutamine, lactate, and rh-tPA concentrations

The concentration of glucose, glutamine, glutamate, and lactate was measured with a YSI 2900 (YSI Life Sciences, Yellow Springs, OH, USA). The concentration of rh-tPA in the culture supernatants was measured by ELISA using a monoclonal goat anti-human tPA antibody (Abcam, Cambridge, UK) and an anti-goat IgG (H+L) secondary antibody conjugated to horseradish peroxidase (Pierce ThermoFisher, Carlsbad, CA, USA, cat. 31430), which was revealed with *o*-Phenylenediamine dihydrochloride (Sigma-Aldrich, Hamburg, Germany). The reaction color was measured at 450 nm (Thermo Scientific, Multiskan FC, UK), and the rh-tPA was quantified with a calibration curve using Actilyse® as a standard (Boehringer Ingelheim, Ingelheim am Rhein, Germany). The rh-tPA productivity (*q*_tPA_) was calculated using Eq 1.

qtPA=(pgcellday)=[finaltPAconcentration−intialtPAconcentrationfinalcellconcentration−initialcellconcentration]×μ(day−1) (1)

### RNA isolation and library preparation

Duplicate samples for the transcriptome analysis were taken 24 h and 48 h after TDS. The control RNA was collected at 48 h from cultures grown at 37°C. RNAlater® (Thermo Fisher Scientific, Waltham MA, USA) was added to preserve RNA in each sample. Total RNA was isolated from 1 × 10^7^ cells using an RNeasy kit (Qiagen, Germantown MD, USA) and quantified on a NanoDrop 1000 spectrophotometer (ThermoFisher Scientific, Waltham, MA, USA). RNA integrity was verified using an Agilent 2100 Bioanalyzer system (Agilent Tech., Santa Clara, CA, USA). The RNA-seq libraries from mRNA samples were prepared from total RNA using an Illumina^®^ TruSeq RNA Sample Preparation Kit (Illumina, San Diego, CA, USA). mRNA sample preparation was performed using an mRNA-Seq Sample preparation kit (Illumina, San Diego, CA, USA).

### Transcriptome sequencing

The mRNA samples were sequenced using an Illumina GAIIX platform with two different configurations (GAIIX, Illumina Inc., San Diego, CA, USA). The first configuration corresponded to 36 bp, with single reads for 48-h (control) and 72-h (24 h after TDS, biphasic) samples. The second sequencing configuration corresponded to 72 bp, with paired-end reads for samples collected at 48 h (control) and 96 h (48 h after TDS, biphasic). All libraries were prepared and performed with one biological replicate.

### Bioinformatic analysis

After quality control check and adapter removal, the Illumina reads for each condition were mapped to the CHO-K1 reference genome published on www.chogenome.org [28], using the short-read aligner, SMALT (http://www.sanger.ac.uk/resources/software/smalt/) v7.4. Two libraries were prepared from biological replicates: the first contained samples from 48 h and 72 h (24 h after TDS) of culture, and the second contained samples from 48 h and 96 h (48 h after TDS) of culture. The read counts for each coding DNA sequence (CDS) in each condition were analyzed with the non-parametric and data-adaptive algorithm NOISeq R package [29], following TMM normalization (S1 Fig). The resulting differentially expressed genes were analyzed using the topGO algorithm in the R package for enrichment analysis of gene ontology [30] (topGO: Enrichment Analysis for Gene Ontology, Bioconductor package version 2.6.0).

### Validation of transcriptional analysis by real time-PCR

Total RNA from samples collected at 48 h (control) and 96 h (48 h after TDS) was extracted using an RNeasy Mini Kit (Qiagen, Hilden, Germany). The RevertAid kit (Fermentas, ThermoFisher, Waltham, MA, USA) was used to obtain cDNA. For PCR detection, we used SYBR Green PCR Master Mix (Applied Biosystems, USA). Reactions of 12 μL were evaluated, containing 6 μL of SYBR green PCR master mix (Applied Biosystems, CA, USA), 150 nM primers, and 50 ng of template cDNA. The reaction was incubated at 50°C for 2 min, 95°C for 10 min, and for 40 cycles at 95°C for 15 s and 60°C for 1 min. Amplification was carried out in a PikoReal 24 Real-Time PCR System and analyzed with PikoReal software 2.2 (ThermoFisher, Waltham, MA, USA). All samples were run in triplicate, and negative controls were included. Data was analyzed using the 2-∆∆Cq method [31] and normalized using *Gst1* as an endogenous control. The primers used are listed in Table 1.

**Table 1:** List of primer sequences used for verification of differentially expressed genes determined by NGS.

PrimerGen ID	Forward (5′-3′)	Reverse (5′-3′)	Amplicon (bp)
***Rbm3***	ATGACAGGCACTTGAAGACC	CCCCGGGATCTTTGAGTCTC	90
***Cirp***	AGTGAGCACCTTGTCCCCAC	AAAGTCTGGTCACGAGGCCA	91
***Ldha***	GTCCTAGGGGAGCATGGAGA	TTGTCGGTATCAGTGCCCAG	107
***rh-tPA***	GAGGAAGGAAATTGCAGGGC	GGCTCTATGTCGGAAGATTT	91
***Sec61***	GTTGGCCCTGTTCCAGTGTT	GCAGCCAAATCTATGAGC GT	100
***Gstp1***	TGGTGAATGATGGGGTGGAA	GGCAGGGCCTTCACATAGTC	97
***Actinb***	AGCTGAGAGGGAAATTGTGCG	AGGAAGAGGATGCAGCAGTG	95
***Neu2***	CAAGACGGATGAGCATGCAG	CACCTCCTCAGCTTGCCACT	91
***Pdia3***	GAGGCTTGCCCCTGAGTATG	GGTGTTTGTGTTGGCAGTGC	91
***Gadph***	AGAAGGTGGTGAAGCAGGCAT	CAAAGGTGGAAGAGTGGGAGTC	87

### Statistical analysis

Kinetics and stoichiometric parameters in biphasic and control cultures (growth, viability, consumption of glucose and glutamine, lactate production, and recombinant protein production) were calculated from at least three independent experiments and are expressed as mean ± standard deviation. The Student´s *t*-test was employed to determine significant differences between the control (37°C) and low temperature treatment (30°C), using a significance level of p < 0.05.

## Results

### Effect of TDS on cell growth, glucose consumption, and rh-tPA productivity

**Fig 1:**
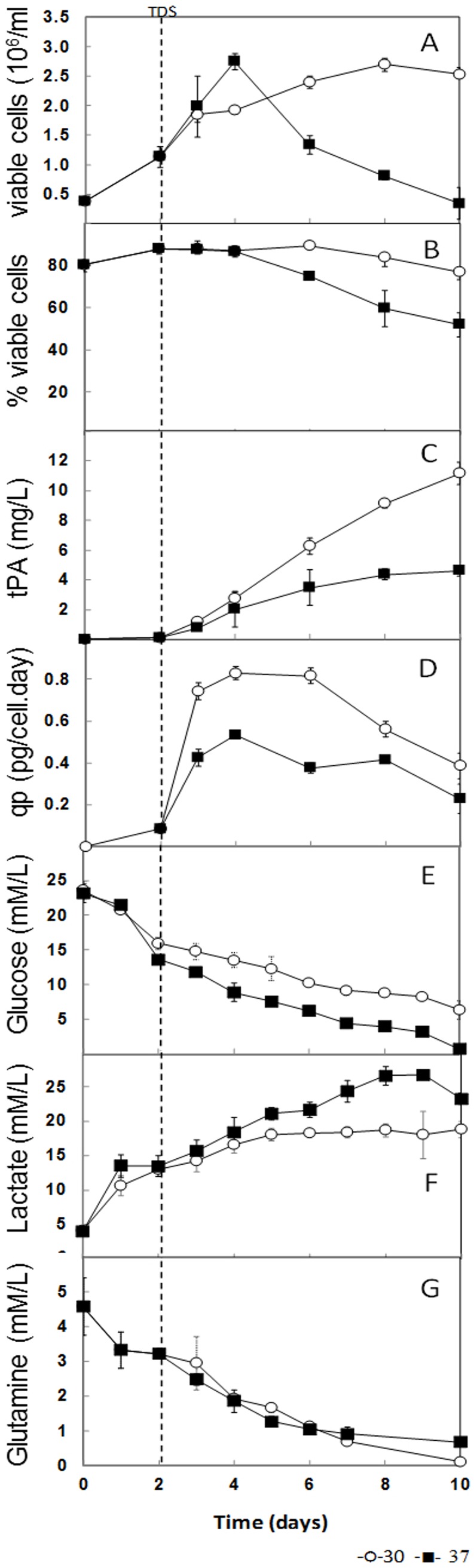
Kinetics of cell growth and rh-tPA production. Recombinant CHO TF70R viable cell density **(A)**, percentage of cell viability **(B)**, rh-tPA production **(C)**, cell-specific rh-tPA production **(D)**, glucose consumption **(E)**, lactate production **(F)**, and glutamine consumption **(G)** of cell cultures at 37°C (closed squares), and under temperature downshift (TDS) to 30°C at 48 h (open circles). Dotted line denotes TDS from 37 to 30°C, and transcriptomic samples were taken at 24 and 48 h after TDS.

CHO TF70R cells that were subjected to moderate hypothermia showed affected growth, viability, and recombinant protein production (Fig 1A–1D). The specific growth rate (μ) before TDS was 0.023 ± 0.012 h^-1^, which decreased significantly to 0.005 ± 0.001 h^-1^ (p < 0.05) after TDS. The maximum viable cell concentration was reached in control cultures (37°C) after 4 days (2.70 ± 0.15 × 10^6^ cells/mL), whereas in biphasic cultures (TDS from 37°C to 30°C at 48 h of culture) similar concentration was obtained only after 8 days (2.67 ± 0.10 x 10^6^ cells/mL) (Fig 1A). Cell viability in biphasic cultures remained above 75% for ten days, whereas in control cultures, it decreased to around 20% (Fig 1B). Similar differences between control and biphasic cultures with respect to cell growth and viability have been previously reported [4,13,27,32], which have been related to the arrest of the cell cycle in the G1/G0 phase and avoidance of apoptosis [4,5,13,33,34].

A maximum rh-tPA concentration of 11.15 ± 0.40 mg/L and maximum *q*_tPA_ of 0.82 ± 0.03 pg/cell-day were obtained in biphasic cultures, which represented a 2.2- and 1.6-fold increase, respectively, as compared to control cultures (Fig 1C and 1D). Such increases in rh-tPA concentration and productivity are similar to results reported by Hendrick *et al*. [27] who reached a rh-tPA concentration of approximately 13 mg/L after 10 days of culture. Moreover, productivity was similar to that reported by Vergara *et al*. [35], with productivity of approximately 0.50 pg/cell-day at 37°C and 0.65 pg/cell-day at 33°C.

Glucose and glutamine concentrations are shown in Fig 1E and 1G, respectively, while lactate concentrations are shown in Fig 1F. Biphasic cultures had 14% lower specific consumption rates of glucose (*q*_glc_) than control cultures (Table 2), resulting in a longer availability (at least 4 more days) of the carbon source. The lactate production rate (*q*_lac_) in biphasic cultures was 19.6% lower than in the control cultures, while the glutamine consumption rate (*q*_gln_) was similar under both conditions (Table 2). A similar *q*_gln_ indicated that pyrimidine synthesis and amino acid synthesis were similar in both culture strategies [36], and that glutamine transport was not substantially affected by TDS. Most likely, TDS increased the amount of glutamine that was converted to protein, while a minor portion was deaminated. Furthermore, the lactate yield from glucose (Y_lac/glc_) was about 19% lower after TDS than in 37°C cultures (Table 2). These data show that TDS results in lower glucose consumption and lactate production as compared to cultures at 37°C. Similar information has been previously reported [3,37,38]. In fact, moderate hypothermia decreases the specific consumption rate and specific production rate of by-products in a cell line-dependent manner [8,13,34].

**Table 2:** Kinetic and stoichiometric parameters of CHO cells in the biphasic and control cultures.

**Parameters**	**Control 37°C**	**Biphasic 30°C**
μ (h^-1^) *	0.021 ± 0.02	0.005 ± 0.001^a^
X_max_ (10^6^ cell/ml)	2.70 ± 0.15	2.67 ± 0.10
Cell viability (%)	87.60 ± 1.50	89.60 ± 2.00
Y_lac/glc_ (mol/mol)	0.85 ± 0.01	0.69 ± 0.02^a^
*q*_tPA_ (pg/cell-day)	0.53 ± 0.02	0.82 ± 0.03^a^
rh-tPA max (mg/L)	4.60 ± 1.20	11.15 ± 0.40^a^
*q*_gln_ (nmol/10^6^ cell h)	26.9 ± 0.81	25.9 ± 1.24
*q*_glc_ (nmol/10^6^ cell h)	112 ± 1.67	98.4 ± 3.54^a^
*q*_lac_ (nmol/10^6^ cell h)	160 ± 1.23	130 ± 7.32^a^

*The specific growth rate (μ) was calculated for the first 4 days in those cultures carried out at 37°C; for biphasic cultures, μ was calculated for the 4 days after temperature downshift.

^a^Biphasic culture data with statistically significant differences (Student’s t-test with a significance level of p < 0.05) from the control culture (37°C) data.

### Differential expression after TDS

**Fig 2:**
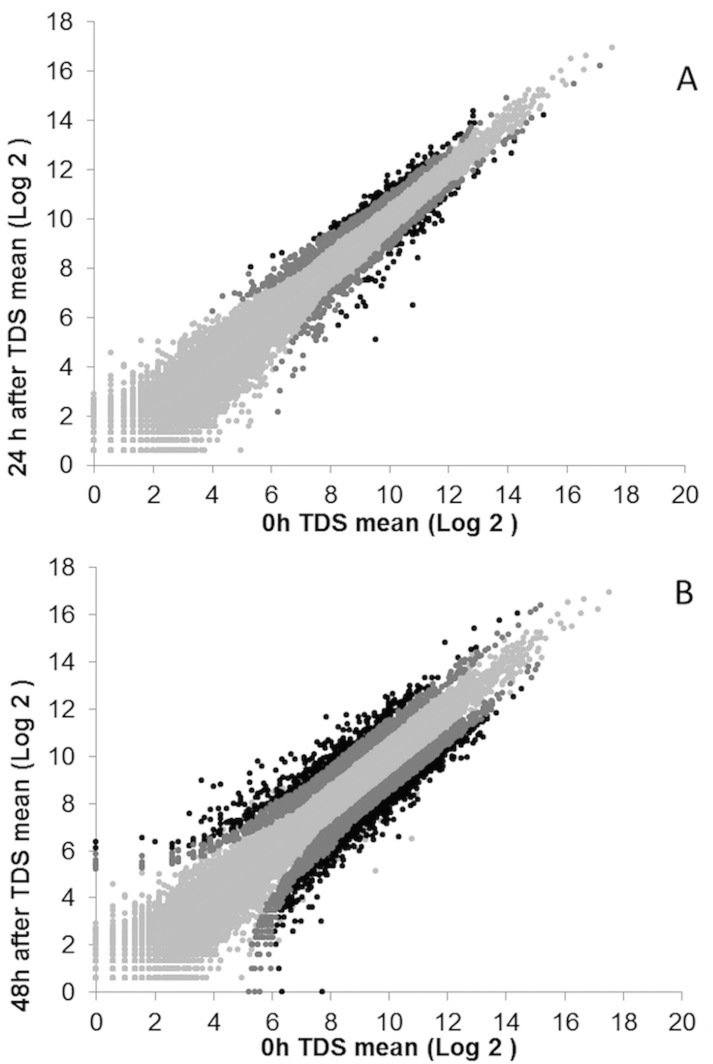
Transcript abundance analysis of 21,789 genes after temperature downshift (TDS). Differential expression determined by Illumina sequencing of duplicated samples collected at 24 h **(A)** and 48 h **(B)** after TDS of culture and compared to the control culture at 37°C, sampled at 48 h of culture (bioinformatic analysis in Materials and Methods). Coding DNA sequence data in each condition were analyzed with the non-parametric and data-adaptive algorithm NOISeq R package [29], following TMM normalization. Genes without differential expression are represented by light gray dots. Genes with differential expression (>1.6 fold change) are shown in dark gray with q of 0.6–0.8 **(A)**, and q of 0.9–0.95 **(B)**. Those that are differentially expressed (>1.6 fold change) with q > 0.8 **(A)** and q > 0.95 **(B)** are shown in black dots.

**Fig 3:**
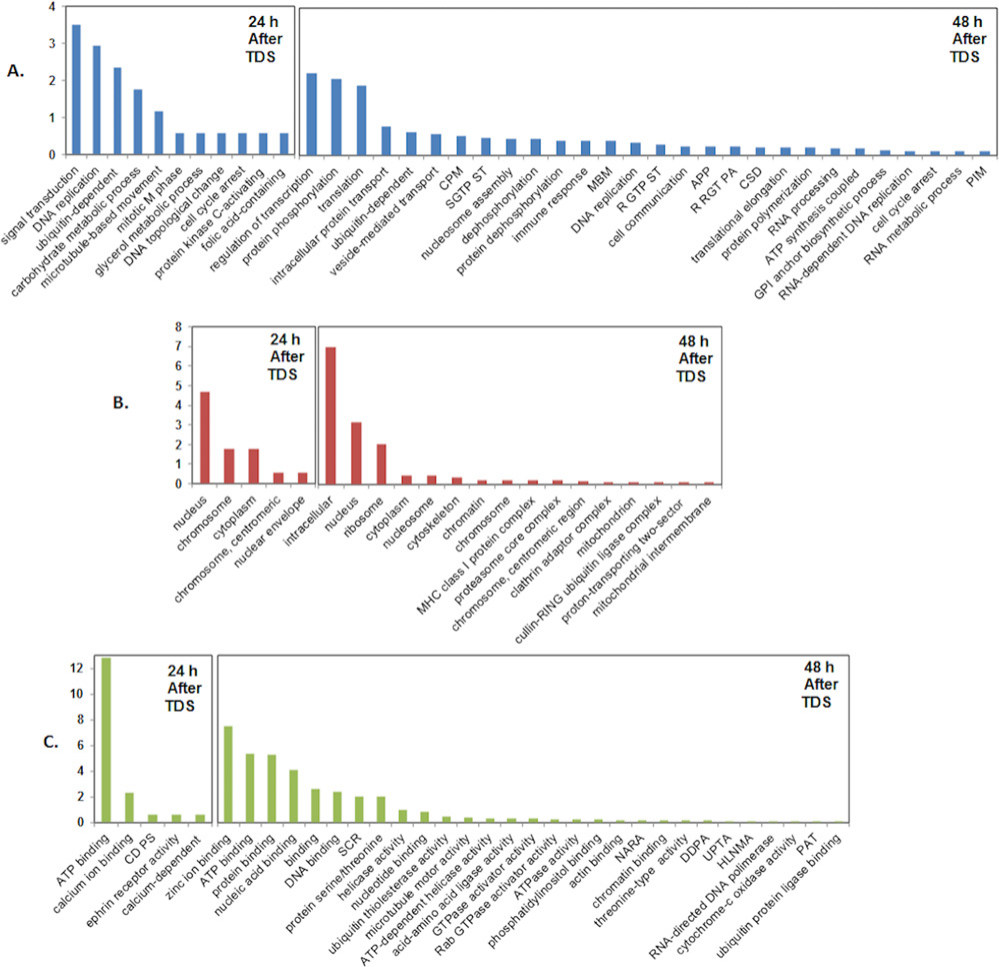
Enriched gene ontology annotation of differentially expressed genes. In total, 171 genes at 24 h after TDS (>1.6 fold change, q > 0.8) and 995 genes at 48 h after TDS (>1.6 fold change, q > 0.95) were classified in sub-ontologies. Enrichment scores on the y-axis and x-axis bar charts represent the follows: GO type—A, Biological process; B, Cellular component; and C, Molecular function. (CPM) cellular protein modulation, (SGTP ST) small GTPase signal transduction, (MBM) microtubule-based movement, (R GTP ST) regulation of GTPase signal transduction, (APP) antigen process and presentation, (R RGT PA) regulation of Rab GTPase activity, (CSD) chromatin assembly or disassembly, (CD PS) cyclin-dependent protein serin.

In total, 21,789 transcripts were mapped, and we annotated 19,775 genes based on the CHO-K1 reference genome (www.chogenome.org [28]). A total of 416 (q > 0.8) and 3,472 (q > 0.9) transcripts with more than a 1.6-fold change were determined to be differentially expressed at 24 and 48 h after TDS, respectively. The normalized expression levels and fold changes of each biological replicate are shown in Fig 2. The differential transcription data was analyzed using the topGO algorithm for GO term enrichment. This analysis grouped 1,421 genes in 57 biological processes categories (Fig 3A). In addition, 568 genes were assigned to 27 cellular component categories (Fig 3B). In the molecular function level, 609 genes were assigned, and they clustered in 50 groups (Fig 3C). As shown in Fig 3, TDS caused most gene changes in the ontological group of biological process, which included signal transduction, DNA replication, transcription, and translation.

Protein -coding genes with >1.6 fold change were clustered in eight groups and are presented in S1 Table. In total, 241 protein-coding genes with q ≥ 0.6 were obtained at 24 h after TDS, and 638 protein-coding genes with q ≥ 0.95 were obtained at 48 h after TDS (S1 Table). When the discussion requires it, we present protein-coding genes with smaller q but whose changes are ≥1.6 fold.

### RNA-seq transcriptome validation by real-time PCR

Although RNA-seq studies are sensitive and specific, we analyzed 11 genes by real-time PCR (RT-PCR) to validate our transcriptome data, as has been done in previous studies [39,40]. To determine a reference gene, different markers (*gst1*, *Gapdh*, *α-tubulin*, and *ß-actin)* were analyzed. Our results indicated that *gst1* was the most stably expressed reference gene in CHO TF70R cells under TDS conditions (Table 3). Genes quantified by RT-PCR included *Rbm3* and *Cirp* as controls for the TDS response, and *rh-tPA* and *Pdia3* as control genes demonstrating no change. In addition, the expression of the *Ldha*, *Sec61*, and *Neu2* genes was analyzed by RT-PCR to verify the overexpression shown in the transcriptomic data. Table 3 shows a comparison of the genes analyzed by RT-PCR and using RNA-seq data. The difference in gene expression using both techniques was less than 30%, thereby validating our results.

**Table 3:** Comparison of fold-changes in differentially expressed genes determined by NGS and by RT-PCR.

Gene	Fold change NGS 48 h after TDS (q)	Fold change real time PCR 48 h after TDS
***Rbm3***	13.54 (0.99)^a^	14.01 ± 0.55^a^
***Cirp***	7.76 (0.99)^a^	7.38 ± 0.28^a^
***rh-tPA***	1.13 (0.34)	1.08 ± 0.08
***Sec61***	2.27 (0.92)^a^	1.57 ± 0.23^a^
***Ldha***	3.15 (0.95)^a^	2.38 ± 0.18^a^
***Gst1* (house keeping)**	1.12 (0.54)	1.01 ± 0.02
***Pdia3***	1.44 (0.72)	1.48 ± 0.16^a^
***Neu2***	3.78 (0.93)^a^	4.42 ± 0.06^a^

^a^Biphasic culture data with statistically significant differences from that of the control culture (37°C).

## Discussion

TDS (from 37°C to 30°C) after 48 h of culture affected the growth of CHO TF70R cells, but extended their cultivation time and maintained higher cell viability for up to 7 days longer than those cultures that were maintained at 37°C. This resulted in a 2.3-fold and 1.6-fold increase in rh-tPA volumetric and specific productivity, respectively, with a concomitant decrease in glucose consumption and lactate production in TDS cultures as compared to cultures maintained at 37°C. These results are consistent with previous reports that used CHO cell lines that were grown under similar conditions [3,4,27] and that suggest that TDS increases recombinant protein production by impacting cell cycle arrest [9,27], increasing mRNA stability [3,4,12], and modulating protein secretion pathways [18]. Here, a transcriptome analysis of CHO TF70R cells was successfully performed, comparing cells grown at 37°C in the exponential phase (48 h of culture) with two time-points after TDS (24 and 48 h). Discussed genes involved in TDS response are summarized in Table 4.

**Table 4:** Comparison of the fold change of highlighted differentially expressed of genes affected by the TDS response. Differentially expressed transcripts (> 1.6 fold) with respect to the control q > 0.8 at 24 hour and q > 0.95 at 48 hour after TDS are indicated in bold lettering. Gene descriptions and putative functions were obtained from www.genecards.org.

Gene	Gene description	Putative function	Fold change NGS, 24 h after TDS (q)	Fold change NGS, 48 h after TDS (q)
*Rbm3*	RNA Binding Motif Protein 3	Cold-inducible mRNA binding protein, enhances protein synthesis at mild hypothermic temperatures	**5.81 (0.95)**	**13.54 (0.99)**
*Cirp*	Cold Inducible RNA Binding Protein	Protective role by stabilizing transcripts of genes involved in cell survival.	**3.31 (0.89)**	**7.76 (0.99)**
*Vim*	Vimentin	Intermediate filament protein attached to the nucleus, ER, and mitochondria	1.47 (0.50)	2.37 (0.93)
*Trpv3*	Transient Receptor Potential Cation Channel, Subfamily V, Member 3	It is activated by innocuous (warm) temperatures	-1.5 (0.07)	1.00 (0.22)
*Dhfr*	Dihydrofolate Reductase	Catalyzes an essential reaction for de novo glycine and purine synthesis	1.14 (0.23)	-1.08 (0.40)
*rh-tPA*	Plasminogen Activator Tissue	Recombinant gene	1.03 (0.09)	1.13 (0.34)
**Cell cycle**				
*Atm*	Serine/threonine protein kinase	Important cell cycle checkpoint kinase, regulator of a wide variety of downstream proteins	-2.16 (0.69)	**-7.49 (1.0)**
*Atr*	Serine/Threonine-Protein Kinase	Related to ATM, cell cycle checkpoint gene required for cell cycle arrest	-1.72 (0.5)	-2.67 (0.94)
*Mdm2*	E3 Ubiquitin Protein Ligase	Mediates ubiquitination of p53/TP53, leading to its degradation by the proteasome	**2.25 (0.85)**	1.65 (0.76)
*Tp53inp2*	Tumor Protein P53 Inducible Nuclear Protein 2	Dual regulator of transcription and autophagy	**5.56 (0.83)**	**4.07 (0.95)**
*Cdkn1a*	Cyclin-Dependent Kinase Inhibitor 1A	Functions as a regulator of cell cycle progression	**3.26 (0.90)**	**9.15 (0.99)**
*Ccnd1*	Cyclin D1	Regulates the cell-cycle during G(1)/S transition	0.03 (0.10)	1.98 (0.79)
*Cdk3*	Cyclin-Dependent Kinase 3	Promotes entry into S phase	-1.07 (0.09)	2.66 (0.66)
*Cdkn2aP16ink4a*	Cyclin-Dependent Kinase Inhibitor 2A	Inducing cell cycle arrest in G1 and G2 phases	1.51 (0.53)	**3.23 (0.95)**
*Tspyl2Cinap*	TSPY-like/SET/nucleosome assembly protein-1	Chromatin remodeling and as an inhibitor of cell-cycle progression	**2.13 (0.82)**	-1.41 (0.71)
*Mybl1*,*Amyb*	V-Myb Avian Myeloblastosis Viral Oncogene Homolog-Like 1	Strong transcriptional activator; involved in the proliferation and/or differentiation	-2.23 (0.56)	**-3.75 (0.95)**
*Mybl2*,*Bmyb*	V-Myb Avian Myeloblastosis Viral Oncogene Homolog-Like 2	Transcription factor involved in the regulation of cell survival, proliferation, and differentiation	**-2.99 (0.91)**	-1.42 (0.72)
*Hspa8*,*Hsc70*	Chaperone Heat Shock 70 kDa Protein 8	Increasing cell survival, repressor of transcriptional activation, participates in the ER-associated degradation	-1.85 (0.66)	-1.48 (0.73)
*Set*	SET Nuclear Proto-Oncogene	Inhibits acetylation of nucleosomes, especially histone H4, by histone acetylases	-1.56 (0.52)	**-3.25 (0.95)**
*Birc6*,*Kiaa1289*	Baculoviral inhibition of apoptosis protein repeat Containing 6	Anti-apoptotic protein which can regulate cell death by controlling caspases	-1.0 (0.13)	**-3.17 (0.96)**
*Brca2*,*Fancd1*	Breast Cancer 2 Tumor Suppressor	Involved in maintenance of genome stability,	- 2.68 (0.74)	**-5.75 (0.99)**
*Gas2*,*Gas-2*	Growth Arrest-Specific 2	Regulation of microfilament dynamic during both cell cycle and apoptosis	- 3.65 (0.73)	**-3.35 (0.95)**
*Brca1*	Breast Cancer 1, Early Onset; E3 ubiquitin-protein ligase	Plays a role in maintaining genomic stability	**- 3.03 (0.90)**	**-4.10 (0.98)**
*Bard1*	BRCA1 Associated RING Domain 1	Interacts with the BRCA1, involved in DNA repair	- 3.39 (0.76)	**-4.19 (0.98)**
*Fancm*,*Kiaa1596*	Fanconi Anemia Group M Protein	Key reaction in DNA repair.	-4.25 (0.48)	**-4.33 (0.93)**
*Brip1*,*Fancj*	Interacting Protein C-Terminal Helicase 1	DNA-dependent ATPase and 5 to 3 DNA helicase required for the maintenance of chromosomal stability	-2.19 (0.58)	**-5.00 (0.98)**
*BLM*	DNA Helicase, RecQ-Like Type 2	DNA helicase, ATP dependent, involved in DNA replication and repair.	-2.61(0.76)	**-2.36(0.96)**
*Rmi1*	RecQ Mediated Genome Instability 1	Role in the processing of homologous DNA recombination intermediates	-1.48 (0.41)	**-3.65 (0.96)**
*Rbl2*	Retinoblastoma-Like 2, cell cycle regulation interacting with E2F-like transcription factor	Key regulator of entry into cell division. Directly involved in heterochromatin formation by maintaining overall chromatin structure	-1.09 (0.16)	**-3.06 (0.95)**
*Rbl1*	Retinoblastoma-Like 1	Involved in cell cycle regulation, interacting with transcription factor E2F-4 and with cyclin E/A-CDK2 complexes	-2.09 (0.75)	**-3.642 (0.96)**
*Rb1*	Retinoblastoma 1	Key regulator of entry into cell division, promotes G0-G1 transition	-2.09 (0.75)	**-3.87 (0.93)**
**Transcription regulation function**				
*Rasl11a*	RAS-Like, Family 11, Member A small GTPase	Regulator of rDNA transcription; mediated signal transduction	**21.65 (0.93)**	**19.20 (0.99)**
*Myc*	Proto-Oncogene C-Myc	Activates the transcription of growth-related genes	-1.15 (0.24)	**3.39 (0.96)**
*c-Fos*	Murine Osteosarcoma Viral Oncogene Homolog	Transcription factor involved in signal transduction, cell proliferation and differentiation		**7.48(0.99)**
**Cell proliferation promoters**				
*Cyr61*,*Ccn1*	Cysteine-Rich, Anigogenic Inducer, 61	Promotes cell proliferation, chemotaxis, angiogenesis and cell adhesion	1.77 (0.62)	**3.89 (0.95)**
*IL33*	Interleukin 33 chromatin-associated nuclear factor	Acts as a chromatin-associated nuclear factor with transcriptional repressor properties	2.28 (0.58)	**23.05 (0.99)**
*Fhl2*	Four And A Half LIM Domains 2	Molecular transmitter linking various signaling pathways to transcriptional regulation	1.58 (0.55)	**5.94 (0.98)**
**Cell death inhibitors**				
*Bcl2l1*,*Bclx*	Apoptosis Regulator Bcl-X	The longer isoform acts as an apoptotic inhibitor and the shorter form acts as an apoptotic activator.	1.73 (0.48)	1.49 (0.71)
*Bcl2l2*,*Kiaa0271*	Apoptosis Regulator BCL-W BCL2-like 2	Promotes cell survival, suppressing death-promoting activity of BAX.	1.03 (0.08)	1.93 (0.77)
*Bcl2a1*	BCL2-Related Protein A1	Retards apoptosis	2.27 (0.46)	2.13 (0.79)
*Bak1*,*Bak*	BCL2-Antagonist/Killer 1	Accelerates programmed cell death by binding to, and antagonizing the anti-apoptotic action of BCL2	1.4 (0.40)	1.78 (0.78)
*Bid*	BH3 Interacting Domain Death Agonist	Induce apoptosis	1.29 (0.32)	2.20 (0.92)
*Pink1*	Serine/Threonine-Protein Kinase PINK1	Protect against C2-ceramide-induced CAD cell death through the PI3K/AKT pathway	1.54 (0.49)	1.80 (0.78)
*Birc5*,*Api4*	Baculoviral IAP Repeat Containing 5	Inhibitor of apoptosis	1.04 (0.09)	1.77 (0.75)
*Grina*, *Nmdara1*	Transmembrane BAX Inhibitor Motif-Containing Protein 3	Glutamate receptor, potential apoptotic regulator	**2.32 (0.85)**	1.78 (0.79)
**Apoptosis activators**				
*Ppid*	Peptidylprolyl Isomerase D	Accelerate the folding of proteins, involved in apoptosis	-3.22 (0.78)	**-3.26 (0.95)**
*Bub1b*, *Mad3I*	Mitotic Checkpoint Serine/Threonine Kinase B,	Implicated in triggering apoptosis in polyploid cells	**-2.34 (0.81)**	-1.69 (0.77)
*Ifih1*	RNA Helicase-DEAD Box Protein 116	Involved in growth inhibition and apoptosis	-1.45 (0.27)	**-4.56 (0.98)**
**Pro-apoptotic activators**				
*Aen*,*Isg20l1*	Apoptosis Enhancing Nuclease	Exonuclease, mediates p53-induced apoptosis	**2.68 (0.86)**	**6.22 (0.99)**
*Bax*	BCL2-Associated X Protein	Accelerates programmed cell death by binding to, and antagonizing the apoptosis repressor BCL2	1.95 (0.66)	**3.18 (0.95)**
*Phlda3*	Pleckstrin Homology-Like Domain 3	Contributes to p53/TP53-dependent apoptosis	2.01 (0.74)	**6.57 (0.99)**
*Pdcd4*,*Ma3*	Programmed Cell Death Protein 4	Participate in apoptotic process	**2.31 (0.80)**	-1.23 (0.60)
*Tnfrsf12a*,*Fgfrp2*	Tumor Necrosis Factor Receptor Superfamily, Member 12A	Positive regulation of apoptotic process	-1.27 (0.36)	**5.57 (0.98)**
*Zc3h12a*	Zinc Finger CCCH-Type Containing 12A,	Triggers apoptosis	1.88 (0.56)	**4.44 (0.98)**
**Protein synthesis**				
*Larp4*,*Pp13296*	La Ribonucleoprotein Domain Family, Member 4	Stimulates mRNA translation	-2.17(0.63)	**-3.48 (0.95)**
*Larp4*,*D13wsu6*	La Ribonucleoprotein Domain Family, Member 4B	Stimulates mRNA translation	-2.24 (0.78)	**-7.27 (0.99)**
*Eif5*	Eukaryotic Translation Initiation Factor 5	Catalyzes the hydrolysis of GTP bound to the 40S ribosomal initiation complex	-1.94 (0.56)	**-3.42 (0.95)**
*Eif4a2*	Eukaryotic Translation Initiation Factor 4A2	ATP-dependent RNA helicase which is a subunit of the eIF4F complex involved in cap recognition and is required for mRNA binding to ribosome	**-3.49 (0.92)**	**-3.29 (0.95)**
**Secretion via ER**				
*Sec61b*	Sec61 Beta Subunit,	beta-subunit protein of the Sec61 complex (transmembrane channel where proteins are translocated across ER membrane)	1.58 (0.54)	2.27 (0.92)
*Sec61g*	Sec61 Gamma Subunit	gamma—subunit protein of the Sec61 complex (transmembrane channel where proteins are translocated across ER membrane)	1.69 (0.53)	1.96 (0.78)
*Bip*	Heat Shock 70kD Protein 5	Master regulator chaperone	1.90 (0.68)	-1.08 (0.40)
*Uggt1*, *Uggt*	UDP-Glc:glycoprotein glucosyltransferase	Re-glucosylates single N-glycans near the misfolded part of the protein, providing quality control for protein folding in the ER	-1.53 (0.53)	-2.97 (0.94)
*Mogs*,*Gcs1*	Mannosyl-Oligosaccharide Glucosidase	Exohydrolysis of the non-reducing terminal glucose residues in the mannosyl-oligosaccharide Glc(3)Man(9)GlcNAc(2)	-1.73 (0.60)	-1.47 (0.72)
*Calr*	Calreticulin	Calcium-binding chaperone that promotes folding, oligomeric assembly and quality control in the endoplasmic reticulum (ER)	1.69 (0.62)	1.61 (0.76)
*Canx*	Calnexin	Membrane ER chaperone that promotes folding	-1.31 (0.38)	-2.11 (0.92)
*Pdia3*,*Grp58*	Protein Disulfide Isomerase 3	Catalyzes the rearrangement of -S-S- bonds in proteins.	1.42 (0.47)	1.44 (0.72)
*Man1A2*	Alpha-1,2-Mannosidase IB	Involved in the maturation of Asn-linked oligosaccharides, trim alpha-1,2-linked mannose residues from Man(9)GlcNAc(2) to produce Man(5)GlcNAc(2)	**-2.38(0.81)**	-2.48(0.93)
*Ssr3*	Translocon-Associated Protein Gamma Subunit	Part of a complex that regulates the retention of ER resident proteins	-1.47 (0.45)	**-3.00 (0.95)**
*Herpud1*	Homocysteine-Inducible, ER Stress-Inducible, Ubiquitin-Like Domain Member 1	Involved in ubiquitin-dependent degradation of misfolded endoplasmic reticulum proteins.	**2.06 (0.81)**	1.32 (0.67)
*Pmm1*	Phosphomannomutase 1, D-mannose 6-phosphate	Catalyzing the second step in the conversion of fructose-6P to GDP-mannose	2.02 (0.74)	**3.42 (0.95)**
*Porcn*,*Porc*	Protein-Cysteine N-Palmitoyltransferase	Key regulator of the Wnt signaling pathway by mediating the attachment of palmitoleate, a 16-carbon monounsaturated fatty acid	1.61 (0.45)	**4.54 (0.97)**
*Dpm3*	Dolichyl-Phosphate Mannosyltransferase Polypeptide 3	Stabilizer subunit of the dolichol-phosphate mannose (DPM) synthase complex	1.42 (0.37)	**3.34 (0.95)**
*Fut1*	Fucosyltransferase 1	Participates in glycosphingolipid biosynthesis	-1.00 (0.07)	**4.81 (0.96)**
*Neu1*	Sialidase 1	Cleaves terminal sialic acid residues from substrates such as glycoproteins and glycolipids	1.94 (0.67)	2.43 (0.93)
*Neu2*	Sialidase 2	Remove sialic acid residues from glycoproteins and glycolipids.	1.94 (0.67)	**3.78 (0.96)**
*Fuca1*	Alpha-L-Fucosidase I	Involved in the degradation of fucose-containing glycoproteins and glycolipids	1.38 (0.44)	2.00 (0.92)
*Fktn*,*Fcmd*	Fukutin, Glycosyltransferase	Involved in the biosynthesis of the phosphorylated O-mannosyl trisaccharide	-2.89(0.69)	**-3.87 (0.95)**
*Galnt1*	Polypeptide N-Acetylgalactosaminyltransferase 1	Catalyze the transfer of N-acetyl-D-galactosamine to serine and threonine residues	-1.43(0.41)	-2.178 (0.93)
*Galnt7*	Polypeptide N-Acetylgalactosaminyltransferase 7	Catalyze the transfer of N-acetyl-D-galactosamine to serine and threonine residues	-1.68 (0.52)	-2.48 (0.93)
*Galnt13*	Polypeptide N-Acetylgalactosaminyltransferase 13	Catalyze the transfer of N-acetyl-D-galactosamine to serine and threonine residues	-3.33 (0.33)	-2.55 (0.71)
*Slc35a3*	UDP-N-Acetylglucosamine (UDP-GlcNAc) Transporter	Supply UDP-GlcNAc as substrate for Golgi-resident glycosyltransferases that generate branching of diantennary oligosaccharides.	-1.17 (0.18)	**-3.93 (0.95)**
**Metabolism**				
*Ldha*	Lactate dehydrogenase A	Glycolysis, energy pathway	**2.51 (0.82)**	**3.15 (0.95)**
*Ldhc*	Lactate Dehydrogenase C	Glycolysis, energy pathway	**19.66 (0.97)**	**5.64 (0.98)**
*Gapdh*	Glyceraldehyde-3-Phosphate Dehydrogenase	Role in glycolysis and nuclear functions	-1.02 (0.09)	1.00 (0.21)
*Hk1*	Hexokinase 1	Glycolysis and gluconeogenesis, energy pathway	1.01 (0.09)	1.10 (0.44)
*Lct*,*Lph*	Lactase	Lactase activity	**-2.08 (0.81)**	-1.49 (0.73)
*Gls*,*Gls1*	Glutaminase	Catalyzes the hydrolysis of glutamine to glutamate and ammonia	1.52 (0.30)	**2.70 (0.91)**
*Asct2*, *Slc1a7*	Solute Carrier Family 1 (Glutamate Transporter), 7	Transports L-glutamate	-1.12 (0.19)	1.83 (0.79)
*Ppcdc*,*Coac*	Phosphopantothenoylcysteine Decarboxylase	Biosynthesis of coenzyme A (CoA) from pantothenic acid (vitamin B5)	**3.27 (0.82)**	**3.73 (0.96)**
*Pgp*	Phosphoglycolate Phosphatase	Phosphoglycolate phosphatase activity	-1.03 (0.10)	**3.95 (0.95)**
*Galk1*	Galactokinase 1	Major enzyme for galactose metabolism	-1.10 (0.18)	**3.58 (0.96)**
*Ndufb7*	NADH Dehydrogenase (Ubiquinone) 1 Beta Subcomplex, 7	Accessory subunit of the mitochondrial membrane respiratory chain NADH dehydrogenase (Complex I)	1.49 (0.44)	**4.44 (0.98)**
*Ndufs7*	NADH Dehydrogenase (Ubiquinone) Fe-S Protein 7	Core subunit of the mitochondrial membrane respiratory chain NADH dehydrogenase (Complex I)	-1.07 (0.15)	**3.10 (0.95)**
*Cbr2*	Carbonyl Reductase 2	Carbonyl reductase	1.87 (0.41)	**4.58 (0.97)**
*Cbr3*	Carbonyl Reductase 3	Carbonyl reductase	1.70 (0.42)	**4.23 (0.98)**
*Gst1*	Glutathione S-Transferase Pi 1	Conjugation of reduced glutathione to a exogenous and endogenous hydrophobic electrophiles	1.00 (0.21)	1.02 (0.54)

### Response to TDS and its effect on recombinant transcripts

An increase in RBM3 and CIRP transcripts has been associated with a cold response in different cell types [41–43]. CIRP has also been associated with increases in mRNA stability [44], which probably improves recombinant protein production [5]. In agreement with such reports, we corroborated the overexpression of transcripts coding for RBM3 and CIRP at 24 and 48 h after TDS by NGS (Table 4 and S1 Table) and RT-PCR (Table 3). Another consistent response to moderate hypothermia is the upregulation of the *vim* gene, and the overexpression of its coded protein (Vimentin) [16,18,45]. Vimentin is a ubiquitous cytoskeleton intermediate filament (IF) [46] and is related to migratory and wound healing processes [47,48]. Here, *vim* was upregulated at 96 h (2.37-fold; q = 0.93), but its function after TDS is still unknown (Table 4). On the other hand, the gene coding for a temperature-activated ion channel (*trpv3*), which is activated at warm temperatures [49], was maintained at basal levels (Table 4). Thus, it is conceivable that this channel could initiate the hypothermia response.

Transcripts expression of recombinant genes encoding for DHFR and rh-tPA was similar in the biphasic and control cultures, for each gene (Table 4). Although the increase in productivity of recombinant proteins has been associated with increases in rh-tPA mRNA abundance and half-life [3,12], we did not observe differential expression of the gene coding for rh-tPA after TDS, either by RT-PCR or in the RNA-seq analysis (Table 3). This suggests that the increase in rh-tPA under TDS might be due to changes in translational events or posttranslational events or due to differences in turnover [3,12,18].

### Differentially expressed transcripts related to the cell cycle

**Fig 4:**
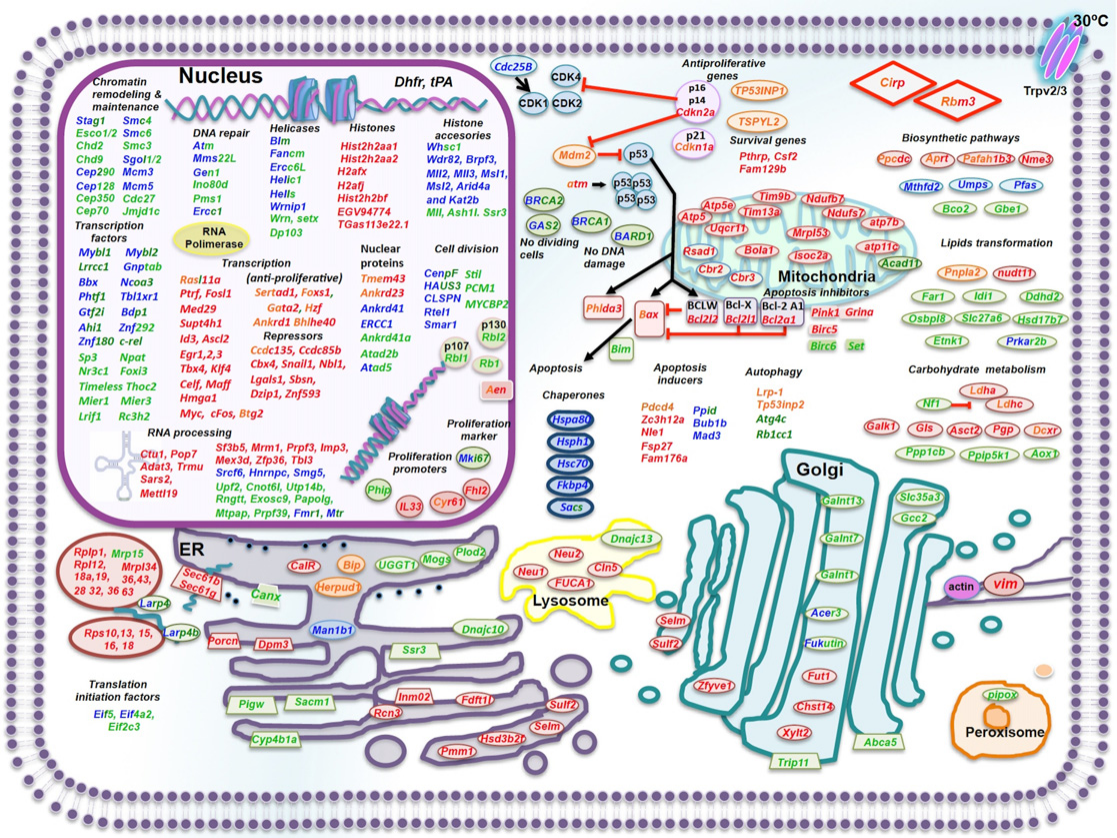
Pictorial representation of transcripts expressed differentially in response to moderate hypothermia after 24 or 48 h of exposition. Differentially expressed genes after 24 h of TDS, up regulated genes are presented in orange; down regulated genes are presented in blue. Differentially expressed genes after 48 h of TDS, up regulated genes are presented in red; down regulated genes are presented in green. Black arrows and red lines represent stimulation and inhibition, respectively.

Up to 89 and 218 cell-cycle genes were differentially expressed at 24 h and 48 h post TDS, respectively. These genes included those related to control of cell cycle progression, transcription, differentiation, and proliferation, among others (S1 Table). Reports indicate that after TDS, different mammalian cells (i.e., glioblastoma cells, fibroblasts, and CHO-K1 cells) initiate cell cycle arrest through p53 activation by the p21 mechanism [50–52]. In fact, CHO-K1 cells exposed to TDS activate the ATR–p53–p21 signaling pathway [52,53]. TDS did not cause differential expression of the gene coding for p53. However, the gene coding for the signal mediator ATM, which participates in p53 phosphorylation [54] and acts in replicating cells [55], was downregulated (Table 4 and S1 Table), as was the gene coding for ATR (ataxia telangiectasia mutated- and Rad3-related kinase), which is related to p53 phosphorylation (2.67-fold; q > 0.94, Table 4). Various studies have described p53 transcriptional regulation of genes like *mdm2*, *TP53INP1*, *waf1/cip1*, and *Bax* [56,57]. After 24 h of TDS, the gene coding for MDM2, which is known to regulate ubiquitination and degradation of p53 [58], and the gene *TP53INP2*, which codes for the antiproliferative tumor protein p53-inducible nuclear protein 1 homologue [59], were overexpressed (Table 4 and S1 Table). TDS caused the overexpression of Cdkn1a (coding for p21) in the two culture timepoints that were studied (Table 4 and S1 Table). p21 is a cyclin-dependent kinase inhibitor that can arrest cell growth through proteins such as CCND1, Cdk2, Cdk3, Cdk4, Cdk6, and probably HSPA8 [60–62]. The overexpression of *Cdkn1a* is consistent with p21 overexpression in CHO-K1 cells after TDS [53], and the effect of p21 has been associated with an inhibition of cell proliferation and increase in recombinant protein productivity [63]. Furthermore, *Cdkn2a*, which encodes for two distinct proteins (p16INK4a and p14ARF) [64,65] involved in cell cycle arrest [66,67], was overexpressed at 48 h after TDS (Table 4 and S1 Table). Thus, the inhibition of cell proliferation and cell cycle arrest due to TDS might be related to the overexpression of *Cdkn1a* and *Cdkn2a*. Moreover, at 24 h after TDS, the gene coding for TSPYL2, a negative cell cycle regulator that could help in arresting cell growth in combination with p21 [68], was upregulated (Table 4 and S1 Table). Additionally, genes coding for MYBL2 and MYBL1, which are transcription factors that promote cell proliferation [69–71], were repressed (Table 4 and S1 Table). *Mybl2* inhibition diminishes proliferation of different cells [72,73], while MYBL proteins can induce transcriptional activation of genes like those that control apoptosis, such as *Api5*, *Birc3*, *Hspa8*, and *Set* [74,75]. In accordance with the repression of *Mybl2* and *Mybl1*, we observed the repression of *Hspa8*, *Set* (TAF1), and *Birc6* (Table 4 and S1 Table). TDS caused (in the two timepoints analyzed) the repression of genes coding for BRCA2 and growth-arrest-specific 2 (GAS2) (Table 4 and S1 Table). The repression of these genes has been associated with non-dividing cells [76,77] and with an anti-apoptotic response through p53 [78,79]. Genes coding for BRCA1 and BARD1, which participate in inhibiting cell proliferation [80,81], were also repressed at both timepoints studied (S1 Table). Interestingly, genes coding for FANCM, BRCA2, FANCJ, BRIP1, BRCA1, BLM, and RMI1, which are overexpressed in disease resulting from genomic instability [82–84], were downregulated after TDS (Fig 4, Table 4 and S1 Table). All these data suggest that TDS promotes cell growth arrest and negatively controls cell proliferation without activating the DNA damage response.

### Differentially expressed transcripts involved in transcription

In this category, 21 genes were differentially expressed at 24 h, and 93 genes were differentially expressed at 48 h after TDS (S1 Table). Interestingly, *Rasl11a*, which codes for a GTPase associated with positive regulation of the transcriptional activity of an RNA polymerase I [85], was highly overexpressed at both time points (21.65- and 19.20-fold, respectively). Moreover, genes coding for the transcription factors Myc and c-FOS, which are involved in growth and cell cycle progression [86,87], were overexpressed at 48 h after TDS (Table 4 and S1 Table). Overexpression of c-FOS in OVCAR8 and SKOV3 cells upregulated the expression of genes involved in *O-*linked and *N-*linked glycosylation, like *FUCA1*, *Man1a1*, *Ganab*, and *Neu1*, and downregulated *Galnt12* and *Galnt14*, among other genes [88]. In this work, TDS caused similar upregulation of *FUCA1* and *Neu1/2* and downregulation of *Galnt1*, *Galnt7*, and *Galnt13* (all with a q > 0.7), which are probably downstream of c-FOS (Table 4 and S1 Table).

### Differentially expressed transcripts related to the delay of cell death

In this group, 8 and 16 genes were differentially expressed at 24 h and 48 h, respectively, of culture after TDS. Genes coding for cell proliferation promoters and antagonists of programmed cell death were overexpressed. Moreover, genes coding for proteins related to apoptotic activation were repressed although the overexpression of pro-apoptotic genes.

TDS causes the overexpression of genes coding for cell proliferation promoters such as Cyr61 (upregulated in both times), alarmin IL33, and Fhl2 (four and a half LIM domains protein 2). In order to avoid apoptosis, genes coding for potent cell death inhibitors such as Bcl-2-like protein 1 (*Bcl-X*) [89] and Bcl-2-like protein 2 (*Bcl2l2*), which mediate cell survival by suppressing the death-promoting activity of BAX [90], were overexpressed at 48 h post TDS (1.49-fold and 1.93-fold, respectively; q > 0.7, Table 4). The mechanism that increases Bcl-xL, which is an isoform of Bcl-X, avoids the translocation of BAX, but has been related to the cold preservation of cardiac tissues [91]. Furthermore, the gene *Bcl2a1*, which codes for Bcl-2-related protein A1 and can retard apoptosis and maintain cell survival by interaction with BAK1, BID, BMF, BCL2L11/BIM, and BAX [92,93], was overexpressed at 48 h after TDS (2.1-fold; q 0.79, Table 4). Likewise, gene coding for PINK1, which can protect against C2-ceramide-induced CAD cell death through the PI3K/AKT pathway [94], and BIRC5 [95], which can increase cell proliferation and prevent apoptosis, were overexpressed at 48 h post TDS (1.8- and 1.7-fold, respectively; q > 0.75, Table 4). In addition, the gene *Grina*, which codes for an apoptotic regulator and controls cell death [96], was overexpressed after 24 h of TDS (Table 4 and S1 Table). Furthermore, genes coding for proteins related to apoptosis activation were downregulated, as was the gene coding for peptidyl-prolyl cis-trans isomerase D (PPID) (downregulated at both time points), the gene coding for Bub1B (downregulated at 24 h), and Ifih1 (downregulated at 48 h after TDS) (Table 4 and S1 Table).

In contrast, genes coding for pro-apoptotic proteins (Aen, Bax, and Phlda3) were upregulated at both time points analyzed (Table 4 and S1 Table). Moreover, the gene that codes for the programmed cell death protein 4 (Pdcd4) was upregulated at 24 h, and the genes coding for the induction of apoptosis, such as tumor necrosis factor receptor (Tnfrsf12a), ribonuclease, and ZC3H12A, were upregulated at 48 h (Table 4 and S1 Table).

Since anti- and pro-apoptotic genes were upregulated, we suggest a complex interplay in regulatory responses, with the most important signal being the anti-apoptotic response, because the cell culture viability and metabolism were extended.

### TDS differentially regulates transcripts associated with protein synthesis and secretion

TDS has been shown to not only increase the productivity of various heterologous proteins, but also to modify their glycosylation patterns [7,32,97]. Hence, we analyzed the effect of TDS on the transcriptome response associated with protein synthesis (ribosome and translation) as well as with protein secretion. A significant upregulation of genes related to ribosomal biogenesis was observed (3 genes 24 h after TDS, and up to 40 genes 48 h after TDS). Most genes encoding for ribosomal proteins belonging to the Rpl, Mrpl, Mrps, and Rps families were upregulated 48 h after TDS (S1 Table). This contrasts with the data reported by Yee *et al*. [18], who observed a downregulation of seven genes, with three from the same family upregulated at 18 h after TDS. This was probably in response to the cells’ increased transcription of ribosome-biogenesis genes, showing a protein-synthesis capacity that had adapted to TDS over time (up to 48 h after TDS). Genes coding for proteins related to mRNA translation (Larp4, Larp4b, and initiation factors Eif5 and Eif4a2) were downregulated at both timepoints under investigation (Table 4 and S1 Table).

With respect to the secretion via ER, the genes coding for Sec61β and Sec61δ, which are part of the translocation complex [98], were overexpressed (2.26- and 1.96-fold, respectively, 48 h after TDS; q > 0.78, Table 4) and presented similar changes after RT-PCR analysis (Table 3). The gene coding for Bip/Hspa5, the master regulator chaperone that assists in translocation, folding, and stabilization of nascent chains [99], was upregulated (1.9-fold at 24 h post TDS; q = 0.68, Table 4). Different enzymes remodel proteins in the lumen of the ER and participate in *N-*glycosylation. These enzymes include oligosaccharyltransferase, glucosidases I (GIsI) and II (GIsII), and UDP-Glc:glycoprotein glucosyltransferase (UGGT1), which is also a folding sensor in ER quality control (ERQC) [100,101]. After 24 h of TDS *Mogs*, which codes for Glucosidase I, was downregulated (1.73-fold; q = 0.60, Table 4), and after 48 h of TDS, downregulation of the gene coding for UGGT1 (2.97-fold; q = 0.94, Table 4) was observed. Once monoglucosylated glycans are formed, they become ligands for the chaperones calnexin and calreticulin, which can interact with oxidoreductases and protein disulfide-isomerase A3 (ERp57/PDIA3) for the rearrangement of disulfide bonds until the nascent protein reaches its native conformation [101]. An interesting finding was that after 48 h, TDS upregulated the expression of the gene coding for calreticulin (1.6-fold; q 0.76, Table 4), while at the same time repressing the gene coding for calnexin (2.11-fold; q 0.92, Table 4). Meanwhile, the gene coding for PDIA3 remained at a constant value (Tables 3 and 4). Proteins that do not correctly fold are recognized by α-mannosidase I, which trims mannoses, and by enhancing α-mannosidase-like 1 (EDEM), marks them for degradation in the proteasome [102]. After 24 h of TDS, the gene *Man1A2* that codes for α-mannosidase I was downregulated (2.38-fold; q = 0.81, Table 4). Likewise, the gene *Ssr3*, which codes for translocon-associated protein subunit gamma (TRAPG), was repressed after 48 h of TDS (Table 4 and S1 Table). TRAPG regulates the retention of ER proteins and marks misfolded proteins to be degraded [103]. Remarkably, the gene coding for Herpud1 was overexpressed 24 h after TDS (Table 4 and S1 Table), although Herpud1 has been associated with ERQC [104].

Other genes coding for enzymes that participate in posttranslational modifications were also differentially expressed during TDS. The gene *Pmm1*, which codes for the enzyme localized at ER and is involved in GDP-mannose and dolichol-phosphate-mannose synthesis [105], was overexpressed at both time points analyzed (Table 4 and S1 Table). Genes coding for N-palmitoyltransferase (*Porcn*) and dolichol-phosphate mannosyltransferase 3 (*Dpm3*), both from the ER [106,107], were upregulated after 48 h of TDS (Table 4 and S1 Table). At the same time, an upregulation of the gene *Fut1*, which codes for the Golgi enzyme galactoside 2-L-fucosyltransferase, was observed (Table 4 and S1 Table). Coding genes for enzymes related to the modification of protein glycosylation profiles were upregulated 48 h after TDS, including *Neu1* and *Neu2* (2.43- and 3.78-fold, respectively; q > 0.93, Table 4), which code for Sialidases 1 and 2 and participate in the removal of sialic acid residues. Likewise, the gene coding for lysosomal alpha-L-fucosidase (*FUCA1*) was overexpressed at 48 h after TDS (2-fold; q = 0.92, Table 4). In contrast, *Fukutin* (*FKTN*), which codes for a glycosyltransferase that is localized in the Golgi, was downregulated at both timepoints (Table 4 and S1 Table). In addition, genes coding for the enzymes N-acetylgalactosaminyltransferase 1, 7, and 13 (GALNT1, 7, and 13), which transfer an N-acetyl-D-galactosamine to peptides localized at the Golgi, were repressed after 48 h of TDS (2.17-, 2.47-, and 2.55-fold, respectively, q > 0.7, Table 4). The same downregulation was observed for the gene *Slc35a3* (Table 4 and S1 Table), which codes for UDP-N-acetylglucosamine transporter that is crucial in N-glycan branching [108].

The transcriptome data described here may be related to modifications in rh-tPA glycosylation caused by TDS [27, 32, 109]. An increased site-occupancy of t-PA around 4% and 7% were found after TDS at 31°C [109] and 33°C [32], respectively, compared with cultures at 37°C. It has been suggested that a site-occupancy increase is associated with lipid-linked oligosaccharide pools [32], as well as differences in bi-antennary and glycan profiles of other proteins [7]. Moreover, a modification in high mannose structures of the single chain t-PA was observed at 48 h post TDS, relative to the control at 37°C [27]. Related to this, we observed the upregulation of *Pmm1* and *Dpm3*, which code for proteins involved in synthesis and transport of nucleotide sugars and dolichol-linked oligosaccharide biosynthesis. Furthermore, variations in glycosylation could be associated with changes in the expression of genes coding for UGGT1, glucosidase I, α-mannosidase I, and TRAPG, which were all downregulated in this work.

Hendrick et al. [27] observed differential recognition of lectin *Maackia amurensis* agglutinin (MAA) by endogenous glycoproteins produced after TDS, indicating changes in N-acetylneuraminic acid α(2id n In our work, the gene coding for α-2,3-sialyltransferase (ST3Gal1) did not change, but the genes *Neu1* and *Neu2* (coding for sialidases) were overexpressed, which could be related to differences in sialic acid content.

Altogether, the changes observed suggest that TDS caused a positive effect in ribosomal biogenesis and variation in genes involved in posttranslational modification via ER and Golgi (Fig 4). This could cause changes in the glycosylation patterns of proteins and enhance rh-tPA productivity. Information on all of these gene modifications can be helpful for understanding how the glycosylation pattern of a given recombinant therapeutic protein is affected in a particular host cell line under TDS, an important point to consider during the production of therapeutic glycoproteins.

### TDS modifies transcription of genes associated with metabolism

Different studies have demonstrated the relevance of metabolic responses of CHO cells to TDS [7,38,110]. Based on GO term assignment, 77 assembled genes were annotated under “metabolism and energy metabolism.” TDS modified genes related to the glycolytic and glutaminolysis pathways and energy metabolism. After TDS, *Ldha/Ldhc*, which code for glycolytic enzymes such as the L-lactate dehydrogenase (LDH) A/C chain [111], were upregulated at both timepoints (Table 4 and S1 Table). Using RT-PCR analysis, *Ldha* presented similar changes after 48 h (Table 3). During the first 48 h after TDS, the overexpression of such genes may be related to lactate production, similar to the control conditions (Fig 1F). The increase in LDH activity has been observed in cold acclimation of rat skeletal muscle, without changes in hexokinase II and glyceraldehyde-3-phosphate dehydrogenase (GAPDH) proteins levels [112]. In comparison, we observed constant values for the genes *Hk1* and *Gapdh*, which code for those enzymes, respectively. The constant expression of *Gapdh* also was verified by RT-PCR (Table 4). At 24 h post TDS, *Lct*, which codes for lactase (known as ß-galactosidase) and participates in conversion of lactose into galactose and glucose, was down-regulated (Table 4 and S1 Table). With respect to glutaminolysis, the gene *Gls*, which codes for glutaminase and catalyzes the conversion of glutamine into glutamate and ammonia as well as *Asct2*, which codes for a glutamine transporter, were overexpressed 48 h after TDS (2.70- and 1.83-fold, respectively q>0.79, Table 4). It is possible that the overexpression of both genes can compensate for the decreased transporter activity after TDS and can explain why *q*_gln_ remains relatively constant after TDS (Table 2). Other central metabolism genes were affected, including the up-regulation of *Ppcdc* at both timepoints (Table 4 and S1 Table), which codes for a cytoplasmic enzyme that is necessary for acetyl CoA biosynthesis [113]. *Pgp* and *Galk1*, which code for phosphoglycolate phosphatase and galactokinase, respectively, were over-expressed 48 h post TDS (Table 4 and S1 Table).

Interestingly, genes coding for retinoblastoma proteins Rbl2 (p130), Rbl1 (p107) (Table 4), and Rb1 (with q 0.93), which can inhibit cell cycle progression, were repressed after 48 h of the TDS. The loss of function of Rb-1, Rbl1, and Rbl2 through triple knock-outs increase the formation of tricarboxylic acid cycle (TCA) intermediates from glutamine [114], which could be related with TCA cycling, mitochondrial metabolism, and recombinant protein production peaks [115] although, we did not see any differential expression of genes involved in the TCA in the current study.

The differentially expressed transcripts related to energy metabolism in mitochondria showed that all of the genes clustered in this group (ten genes) were up-regulated 48 h after TDS (S1 Table), in agreement with extended cell survival and suggesting high mitochondrial activity (Fig 1). Another four genes (of the five found that had altered expression) involved in NADH metabolism were up-regulated after 48 h of TDS (S1 Table). Like *Ndufb7* and *Ndufs7*, which code for NADH dehydrogenases and are required to transfer electrons from NADH to the respiratory chain [116], and *Cbr2* and *Cbr3*, which code for NADPH carbonyl reductases (Table 4 and S1 Table).

Based on the transcriptomic results, extension of the growth phase in cultures under TDS can be associated with the maintenance of active central metabolism and cellular respiration as well as mitochondrial biogenesis. Altogether, most of the transcriptional changes promote the avoidance of apoptotic cell death, and those changes could be associated with the increase in rh-tPA productivity. Finally, the findings reported in the present study provide an informative transcriptome resource, mainly dealing with genes relevant to extending the culture life combined with the increase of recombinant protein productivity after 24 and 48 h of TDS exposure. In the same sense, we are characterizing the proteomic response and the relationship between glycosylation and secretory machinery in this biological model. The information presented should advance the understanding of the responses involved in cold adaptation, increase the knowledge of recombinant CHO cell biology, and can be useful for establishing improved bioprocesses for optimizing the quality and productivity of biopharmaceutical proteins.

## Supporting Information

S1 Fig Analysis of differential expression. Transcriptome contigs were aligned with Ensembl, Genome CHO and GenBank using BLAST, an annotation was assigned to each contig. Differential expression was completed using NOISeq algorithm for comparison of samples 48 h (control) and 72 h (24 h after TDS, biphasic) single end (q > 0.8) and 48 h (control) and 96 h (48 h after TDS, biphasic) paired ends (q > 0.9), and then clustering was conducted using the TopGO algorithm.
(DOCX)

S1 Table Gene expression profiles at 24 and 48 h after TDS of CHO cell culture producing rh-tPA. Only protein-coding genes are shown. Eight main groups are presented. Red color indicates low expression levels, and green color indicates high expression levels. The gene names are shown in the middle of the table and the gene accession numbers are presented. Those differentially expressed genes (>1.6 fold change) were enriched by gene ontology annotation at 24 h (171 genes q ≥ 0.8 and 70 genes 0.6 ≥ q < 0.8) and 48 h (638 genes, q ≥ 0.95) after TDS and classified in sub-ontologies.
(DOC)
