# Supplementary material for: Effect of Temperature Downshift on the Transcriptomic Responses of Chinese Hamster Ovary Cells Using Recombinant Human Tissue Plasminogen Activator Production Culture
Source: PLoS One. 2016 Mar 18;11(3):e0151529. doi: 10.1371/journal.pone.0151529 (PMC4798216; doi:10.1371/journal.pone.0151529)
Supplement: S1 Fig — Transcriptome contigs were aligned with Ensembl, Genome CHO and GenBank using BLAST, an annotation was assigned to each contig. Differential expression was completed using NOISeq algorithm for comparison of samples 48 h (control) and 72 h (24 h after TDS, biphasic) single end (q > 0.8) and 48 h (control) and 96 h (48 h after TDS, biphasic) paired ends (q > 0.9), and then clustering was conducted using the TopGO algorithm. (DOCX) [file pone.0151529.s001.docx]

**Effect of temperature downshift on transcriptomic responses of Chinese hamster ovary cells during recombinant tPA production cultures**

Andrea Bedoya-López, Karel Estrada, Alejandro Sanchez-Flores, Octavio T. Ramírez, Claudia Altamirano, Lorenzo Segovia, Juan Miranda-Ríos, Mauricio A. Trujillo-Roldán and Norma A. Valdez-Cruz

**Supplementary figures and data**

**S1 Fig.** **Analysis of differential expression.** Transcriptome contigs were aligned with Ensembl, Genome CHO and GenBank using BLAST, an annotation was assigned to each contig. Differential expression was completed using NOISeq algorithm for comparison of samples 48 h (control) and 72 h (24 h after TDS, biphasic) single end (q > 0.8) and 48 h (control) and 96 h (48 h after TDS, biphasic) paired ends (q > 0.9), and then clustering was conducted using the TopGO algorithm.

**
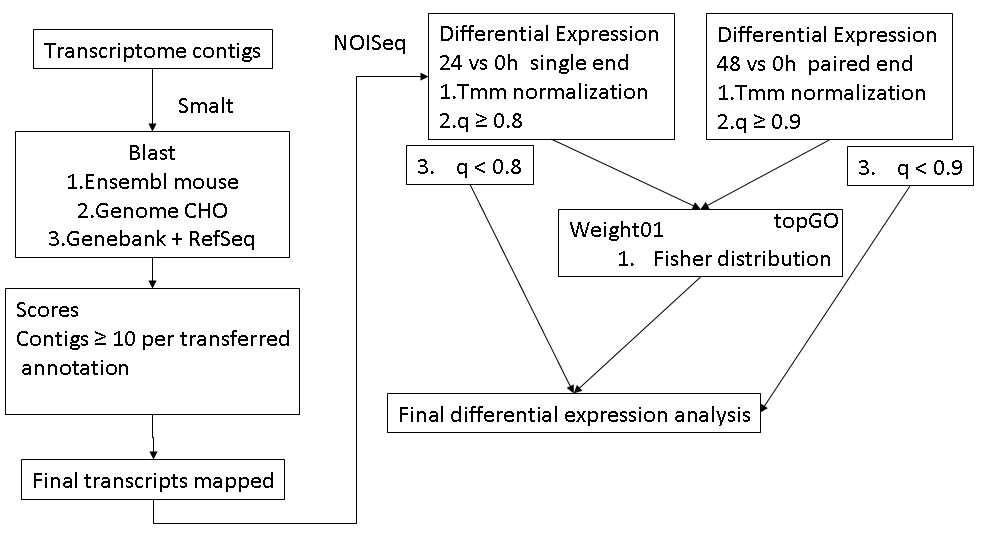
**
