# Supplementary material for: Effect of Temperature Downshift on the Transcriptomic Responses of Chinese Hamster Ovary Cells Using Recombinant Human Tissue Plasminogen Activator Production Culture
Source: PLoS One. 2016 Mar 18;11(3):e0151529. doi: 10.1371/journal.pone.0151529 (PMC4798216; doi:10.1371/journal.pone.0151529)
Supplement: S1 Table — Only protein-coding genes are shown. Eight main groups are presented. Red color indicates low expression levels, and green color indicates high expression levels. The gene names are shown in the middle of the table and the gene accession numbers are presented. Those differentially expressed genes (>1.6 fold change) were enriched by gene ontology annotation at 24 h (171 genes q ≥ 0.8 and 70 genes 0.6 ≥ q < 0.8) and 48 h (638 genes, q ≥ 0.95) after TDS and classified in sub-ontologies. (DOC) [file pone.0151529.s002.doc]

**Effect of temperature downshift on transcriptomic responses of Chinese hamster ovary cells during recombinant tPA production cultures**

Andrea Bedoya-López, Karel Estrada, Alejandro Sanchez-Flores, Octavio T. Ramírez, Claudia Altamirano, Lorenzo Segovia, Juan Miranda-Ríos, Mauricio A. Trujillo-Roldán and Norma A. Valdez-Cruz

**S1 Table.** **Gene expression profiles at 24 and 48 h after TDS of CHO cell culture producing rh-tPA.** Only protein-coding genes are shown. Eight main groups are presented. Red color indicates low expression levels, and green color indicates high expression levels. The gene names are shown in the middle of the table and the gene accession numbers are presented. Those differentially expressed genes (>1.6 fold change) were enriched by gene ontology annotation at 24 h (171 genes q ≥ 0.8 and 70 genes 0.6 ≥ q < 0.8) and 48 h (638 genes, q ≥ 0.95) after TDS and classified in sub-ontologies.

|  |  | **48/72h** | **48/96h** | **Gene name** | **GenBank Accession** | **q 48/72h** | **q 48/96h** |  | **Color scale** |
| --- | --- | --- | --- | --- | --- | --- | --- | --- | --- |
| **Cell cycle** | **Cell cycle** | 3.26 | 9.15 | Cdkn1a,Cip1,Waf1 | EGW12095 | 0.91 | 1.00 | **≥** | **5.00** |
|  |  | -2.16 | -7.49 | Atm | EGW14189 | 0.69 | 1.00 |  | **4.00** |
|  |  | -2.68 | -5.75 | Brca2,Fancd1 | EGV98879 | 0.75 | 0.99 |  | **3.00** |
|  |  |  | -5.28 | N4bp2 | EGW09169 |  | 0.99 |  | **2.00** |
|  |  |  | 4.53 | Sac3d1 | EGW12257 |  | 0.98 |  | **1.00** |
|  |  | -2.22 | -4.34 | Cenpf | EGV92559 | 0.79 | 0.98 |  | **0.00** |
|  |  |  | -3.99 | Pcm1 | EGV99922 |  | 0.98 |  | **-1.00** |
|  |  | -2.15 | -4.04 | Haus3 | EGW11038 | 0.65 | 0.98 |  | **-2.00** |
|  |  |  | 5.42 | Rprm | EGW13478 |  | 0.98 |  | **-3.00** |
|  |  |  | -4.37 | Mll5 | EGV97016 |  | 0.98 |  | **-4.00** |
|  |  |  | -4.49 | Lrrcc1 | EGV93499 |  | 0.98 | **≤** | **-5.00** |
|  |  |  | -4.29 | Fign | EGW05697 |  | 0.96 |  |  |
|  |  | -2.12 | -3.71 | Pds5b,Aprin,As3,Kiaa0979 | EGV98876 | 0.79 | 0.96 |  |  |
|  |  | -3.31 | -3.97 | Ercc6l | EGW14035 | 0.86 | 0.96 |  |  |
|  |  | 3.38 | 3.77 | Psrc1 | EGW10029 | 0.89 | 0.96 |  |  |
|  |  | -2.41 | -3.56 | Fignl1,S30 | EGW07731 | 0.68 | 0.96 |  |  |
|  |  | -2.41 | -3.35 | Hells,Lsh,Pasg | EGV98688 | 0.81 | 0.96 |  |  |
|  |  | -3.29 | -3.53 | Cep128 | EGW05822 | 0.77 | 0.96 |  |  |
|  |  |  | -3.29 | Mycbp2,Kiaa0916,Pam | EGW02134 |  | 0.96 |  |  |
|  |  |  | 3.24 | Cdkn2a | EGV95216 |  | 0.95 |  |  |
|  |  |  | -2.99 | Stil | EGV94551 |  | 0.95 |  |  |
|  |  |  | 3.13 | Pelo | EGV92177 |  | 0.95 |  |  |
|  |  |  | 3.07 | Gadd45gip1 | EGW00619 |  | 0.95 |  |  |
|  |  |  | -3.41 | Taf1 | EGW15071 |  | 0.95 |  |  |
|  |  | -3.65 | -3.35 | Gas2,Gas-2 | EGV94150 | 0.73 | 0.95 |  |  |
|  |  |  | 3.00 | E4f1 | EGW09743 |  | 0.95 |  |  |
|  |  |  | 2.18 | E4f1 | EGW09739 |  | 0.95 |  |  |
|  |  |  | -3.49 | Mitd1 | EGW10903 |  | 0.95 |  |  |
|  |  |  | -3.36 | Sass6 | EGW02968 |  | 0.95 |  |  |
|  |  |  | 3.85 | Fbxl15 | EGV98257 |  | 0.95 |  |  |
|  |  | -2.60 |  | Clspn | EGW01090 | 0.82 |  |  |  |
|  |  | -2.28 |  | Rtel1 | EGW11083 | 0.82 |  |  |  |
|  |  | 2.28 |  | Anxa1,Anx1 | EGW02515 | 0.85 |  |  |  |
|  |  | 3.38 |  | Psrc1,Dda3 | EGW10029 | 0.89 |  |  |  |
|  |  | 2.80 |  | Ccng1,Ccng | EGV97646 | 0.84 |  |  |  |
|  |  | -2.63 |  | Banp,Smar1 | EGV94090 | 0.83 |  |  |  |
|  |  | -2.26 |  | Cdc25b,Cdc25m2 | EGV92415 | 0.82 |  |  |  |
|  |  |  |  |  |  |  |  |  |  |
|  | **DNA Repair** | -4.72 | -6.02 | Gen1 | EGW02733 | 0.59 | 0.99 |  |  |
|  |  | -4.72 | -3.69 | Gen1 | EGW02732 | 0.59 | 0.99 |  |  |
|  |  | -3.40 | -4.19 | Bard1 | EGW07526 | 0.76 | 0.98 |  |  |
|  |  | -2.74 | -4.03 | Fancm,Kiaa1596 | EGV96874 | 0.74 | 0.98 |  |  |
|  |  |  | -3.88 | Ino80d | EGW02355 |  | 0.95 |  |  |
|  |  | -3.27 | -3.54 | Mms22l,C6orf167 | EGW02936 | 0.68 | 0.95 |  |  |
|  |  |  | -3.25 | Poli,Rad30b | EGW01126 |  | 0.95 |  |  |
|  |  |  | -3.09 | Setx | EGW12320 |  | 0.95 |  |  |
|  |  | -2.42 |  | Tonsl,Ikbr,Nfkbil2 | EGV92699 | 0.82 |  |  |  |
|  |  | -2.15 |  | Wrnip1,Whip | EGV99238 | 0.81 |  |  |  |
|  |  |  |  |  |  |  |  |  |  |
|  | **Chromatin binding** |  | -4.81 | Esco2 | EGW05781 |  | 0.98 |  |  |
|  |  | -1.94 | -3.81 | Stag1 | EGV96702 |  | 0.96 |  |  |
|  |  |  | -3.39 | Chd2 | EGV94692 |  | 0.96 |  |  |
|  |  |  | -3.03 | Esco1 | EGW07573 |  | 0.95 |  |  |
|  |  |  | -3.06 | Rbl2 | EGV92006 |  | 0.95 |  |  |
|  |  |  | -3.01 | Chd9,Kiaa0308,Pric320 | EGV92004 |  | 0.95 |  |  |
|  |  |  |  |  |  |  |  |  |  |
|  | **Chromosome** | -2.60 | -4.72 | Mphosph9 | EGW14952 | 0.77 | 0.98 |  |  |
|  |  |  | -4.12 | Cep350 | EGW10151 |  | 0.98 |  |  |
|  |  | -2.17 | -3.89 | Smc4,Capc,Smc4l1 | EGW14560 | 0.81 | 0.96 |  |  |
|  |  |  | -4.62 | Kiaa0586 | EGW13522 |  | 0.96 |  |  |
|  |  | -1.83 | -3.41 | Smc6,Kiaa4103,Smc6l1 | EGW02731 | 0.65 | 0.96 |  |  |
|  |  |  | -3.34 | Kiaa1731 | EGV97879 |  | 0.96 |  |  |
|  |  | -2.23 | -3.36 | Sgol2 | EGV92175 | 0.80 | 0.96 |  |  |
|  |  | -2.61 | -3.41 | Blm | EGW13408 | 0.76 | 0.96 |  |  |
|  |  |  | -3.22 | Smc3,Bam,Bmh,Cspg6,Smc3l1 | EGW10414 |  | 0.96 |  |  |
|  |  | -2.07 | -3.23 | Mki67 | EGW11758 | 0.82 | 0.96 |  |  |
|  |  |  | -3.27 | Cep70 | EGV98744 |  | 0.95 |  |  |
|  |  | -2.45 | -3.12 | Sgol1,Sgo1 | EGV96424 | 0.72 | 0.95 |  |  |
|  |  | -2.24 | -3.17 | Cenpk | EGW03513 | 0.61 | 0.95 |  |  |
|  |  |  |  |  |  |  |  |  |  |
|  | **Nucleus** |  | 16.00 | Hist2h2aa1,Hist2h2aa2 | EGV94778 |  | 1.00 |  |  |
|  |  |  | 11.62 | Hist2h2aa1,Hist2h2aa2 | EGV94775 |  | 1.00 |  |  |
|  |  |  | 41.25 | H2afj | EGV96660 |  | 1.00 |  |  |
|  |  |  | 6.96 | EGV94774 | EGV94774 |  | 0.99 |  |  |
|  |  |  | -5.00 | Brip1,Bach1,Fancj | EGV98907 |  | 0.99 |  |  |
|  |  |  | 4.37 | H2afx,H2ax | EGV99105 |  | 0.98 |  |  |
|  |  | 2.01 | 4.06 | Ankrd23 | EGW06453 | 0.61 | 0.97 |  |  |
|  |  |  | 4.24 | EGV94288 | EGV94288 |  | 0.97 |  |  |
|  |  |  | 4.54 | Hist2h2bf | EGV94771 |  | 0.97 |  |  |
|  |  |  | 3.73 | Fam129b | EGW07686 |  | 0.96 |  |  |
|  |  |  | -3.95 | Mll3 | EGV91769 |  | 0.96 |  |  |
|  |  |  | -3.79 | Pms1,Pmsl1 | EGW06544 |  | 0.96 |  |  |
|  |  |  | -3.66 | Cdc27 | EGV96757 |  | 0.96 |  |  |
|  |  |  | -3.75 | Cdc27 | EGW11571 |  | 0.96 |  |  |
|  |  |  | 3.47 | H1f0,H1fv | EGW02232 |  | 0.96 |  |  |
|  |  | 1.73 | 3.41 | Tgas113e22.1 | EGW02037 | 0.63 | 0.96 |  |  |
|  |  | 1.73 | 3.31 | Tgas113e22.1 | EGW02037 | 0.63 | 0.96 |  |  |
|  |  | -2.92 | -3.36 | Whsc1,Kiaa1090,Nsd2 | EGW00396 | 0.89 | 0.96 |  |  |
|  |  |  | -2.04 | Mll2,Alr,Kmt2b | EGW10393 |  | 0.95 |  |  |
|  |  |  | -3.23 | Jmjd1c | EGW00908 |  | 0.95 |  |  |
|  |  | 2.55 | 2.98 | Tmem43 | EGV97713 | 0.86 | 0.95 |  |  |
|  |  |  | -3.41 | S100pbp | EGW00101 |  | 0.95 |  |  |
|  |  | -2.18 | -3.03 | Ints6 | EGW10363 | 0.74 | 0.95 |  |  |
|  |  | -3.31 | 3.21 | Ercc1,Ercc-1 | EGW05346 |  | 0.95 |  |  |
|  |  |  | -3.16 | Arid4b,Sap180 | EGV95692 |  | 0.95 |  |  |
|  |  |  | -3.26 | Rragb | EGW13363 |  | 0.95 |  |  |
|  |  |  | -3.29 | Msl1 | EGW13030 |  | 0.95 |  |  |
|  |  | -2.64 |  | Brpf3,Kiaa1286 | EGW03613 | 0.86 |  |  |  |
|  |  | -3.08 |  | Ankle1,Ankrd41,Lem3 | EGV97491 | 0.85 |  |  |  |
|  |  | -2.29 |  | EGV92912 | EGV92912 | 0.82 |  |  |  |
|  |  | -2.07 |  | Parp1,Adprt | EGW11182 | 0.82 |  |  |  |
|  |  | -2.20 |  | Wdr82,Cdw5 | EGW05670 | 0.80 |  |  |  |
|  |  | 2.14 |  | Tspyl2,Cinap,Dentt,Dxbwg1396e | EGW12451 | 0.82 |  |  |  |
|  |  |  |  |  |  |  |  |  |  |
|  | **Replication** |  | -4.69 | Rev3l,Polz,Sez4 | EGW10119 |  | 0.98 |  |  |
|  |  |  | -3.66 | Rmi1 | EGW07101 |  | 0.96 |  |  |
|  |  | -3.18 | -3.88 | Polq,Polh | EGV92665 | 0.82 | 0.96 |  |  |
|  |  | -2.61 | -2.36 | Blm | EGW13408 | 0.76 | 0.96 |  |  |
|  |  | -2.42 |  | Pold1 | EGW13445 | 0.85 |  |  |  |
|  |  | -2.47 |  | Mcm5,Cdc46,Mcmd5 | EGW03570 | 0.87 |  |  |  |
|  |  | -2.42 |  | Mcm3,Mcmd,Mcmd3 | EGW10037 | 0.86 |  |  |  |
|  |  | 2.33 |  | Obfc1,Stn1 | EGW08413 | 0.83 |  |  |  |
|  |  |  |  |  |  |  |  |  |  |
|  | **Transcription** | 21.65 | 19.20 | Rasl11a | EGV93376 | 0.94 | 1.00 |  |  |
|  |  | 2.42 | 9.74 | Sertad1 | EGW06411 | 0.77 | 1.00 |  |  |
|  |  | -2.42 | -7.18 | Znf292,Kiaa0530 | EGW05057 | 0.69 | 0.99 |  |  |
|  |  | 4.23 | 9.30 | Btg2 | EGW02253 | 0.73 | 0.99 |  |  |
|  |  | 2.26 | 6.55 | Foxs1,Fkh3,Fkhl18,Freac10 | EGW00291 | 0.83 | 0.99 |  |  |
|  |  |  | -8.27 | Znf483,Kiaa1962,Zkscan16 | EGW11192 |  | 0.99 |  |  |
|  |  |  | -6.17 | Znf595 | EGW02064 |  | 0.99 |  |  |
|  |  |  | 7.48 | c-Fos | EGW02797 |  | 0.99 |  |  |
|  |  |  | 14.13 | Id3 | EGW04769 |  | 0.99 |  |  |
|  |  |  | -7.23 | Rel | EGW11604 |  | 0.99 |  |  |
|  |  |  | 6.59 | Cebpd,Celf | EGW06574 |  | 0.99 |  |  |
|  |  |  | 9.74 | Egr2,Egr-2,Krox-20,Krox20 | EGV91557 |  | 0.99 |  |  |
|  |  |  | -5.07 | Mga,Kiaa4252 | EGV97255 |  | 0.99 |  |  |
|  |  |  | 5.39 | Tceb2 | EGv94718 |  | 0.98 |  |  |
|  |  |  | 3.48 | Tceb2 | EGW13825 |  | 0.98 |  |  |
|  |  |  | 2.90 | Tceb2 | EGW10256 |  | 0.98 |  |  |
|  |  |  | -5.83 | Rcor1,D12wsu95e,Kiaa0071 | EGV94872 |  | 0.98 |  |  |
|  |  |  | 4.85 | Ccdc85b | EGV99361 |  | 0.98 |  |  |
|  |  | 3.98 | 8.58 | Ankrd1 | EGW00744 | 0.70 | 0.98 |  |  |
|  |  |  | -2.15 | Znf208,Znf91l | EGW02063 |  | 0.98 |  |  |
|  |  |  | 4.11 | Fosl1,Fra1 | EGV99360 |  | 0.98 |  |  |
|  |  | -1.97 | -4.33 | Bdp1,Kiaa1241,Tfnr | EGW04154 | 0.63 | 0.98 |  |  |
|  |  |  | -4.96 | Rc3h2,Mnab,Rnf164 | EGW04601 |  | 0.98 |  |  |
|  |  |  | -4.37 | Znf518a,Zfp518,Znf518 | EGV99258 |  | 0.98 |  |  |
|  |  |  | -6.39 | Znf827,Zfp827 | EGW00223 |  | 0.98 |  |  |
|  |  | -2.80 | -4.07 | Phtf1 | EGW08739 | 0.82 | 0.98 |  |  |
|  |  |  | -2.43 | Thoc2 | EGW11919 |  | 0.98 |  |  |
|  |  |  | -2.78 | Thoc2 | EGW11920 |  | 0.98 |  |  |
|  |  |  | -4.08 | Thoc2 | EGW14279 |  | 0.98 |  |  |
|  |  |  | -2.77 | Sp3 | EGV95684 |  | 0.98 |  |  |
|  |  |  | -4.46 | Sp3 | EGV92334 |  | 0.98 |  |  |
|  |  |  | -4.01 | Znf160,Kiaa1611 | EGW13760 |  | 0.98 |  |  |
|  |  |  | -2.21 | Znf638,Np220,Zfml | EGW12481 |  | 0.98 |  |  |
|  |  |  | -4.46 | Znf638,Np220,Zfml | EGW12480 |  | 0.98 |  |  |
|  |  |  | -4.22 | Znf182,Znf21 | EGV92942 |  | 0.98 |  |  |
|  |  |  | 6.50 | Egr3,Egr-3 | EGV91970 |  | 0.98 |  |  |
|  |  |  | -4.11 | Zbtb10,Rinzf | EGW09915 |  | 0.97 |  |  |
|  |  |  | 31.00 | Tbx4 | EGV94939 |  | 0.97 |  |  |
|  |  |  | 4.84 | Ascl2,Bhlha45,Hash2 | EGW08475 |  | 0.97 |  |  |
|  |  |  | 4.87 | Maff | EGW06784 |  | 0.97 |  |  |
|  |  |  | -4.89 | Znf329,Zfp329 | EGW09797 |  | 0.96 |  |  |
|  |  |  | 13.00 | Cux2,Cutl2 | EGW07437 |  | 0.96 |  |  |
|  |  | -1.74 | -3.82 | Bbx,Hbp2 | EGV96821 | 0.60 | 0.96 |  |  |
|  |  |  | -3.76 | Npat | EGW14188 |  | 0.96 |  |  |
|  |  |  | 3.90 | Med29,Ixl | EGV93043 |  | 0.96 |  |  |
|  |  | -2.10 | -3.64 | Rbl1 | EGV97973 | 0.75 | 0.96 |  |  |
|  |  |  | -3.56 | Csde1,D3jfr1 | EGW00037 |  | 0.96 |  |  |
|  |  |  | -5.19 | Hnf4a,Hnf4,Nr2a1,Tcf14 | EGW09397 |  | 0.96 |  |  |
|  |  |  | -3.74 | Nr3c1,Grl | EGW10246 |  | 0.96 |  |  |
|  |  |  | 3.85 | Supt4h1,Qtsa-10763 | EGV96618 |  | 0.96 |  |  |
|  |  |  | -3.66 | Zfp60,Mfg3 | EGW06402 |  | 0.96 |  |  |
|  |  |  | 3.39 | Myc | EGW14653 |  | 0.96 |  |  |
|  |  |  | -3.37 | Nfat5,Kiaa0827,Tonebp | EGW04760 |  | 0.96 |  |  |
|  |  |  | -3.58 | Zfp120 | EGW11654 |  | 0.96 |  |  |
|  |  |  | -6.41 | Zfp120 | EGW15131 |  | 0.96 |  |  |
|  |  |  | -4.92 | Zfp120 | EGW12002 |  | 0.96 |  |  |
|  |  |  | -3.12 | Zfp120 | EGW14883 |  | 0.96 |  |  |
|  |  |  | 3.43 | Ptrf | EGW08042 |  | 0.96 |  |  |
|  |  |  | -3.47 | Mier1 | EGV99055 |  | 0.96 |  |  |
|  |  |  | 3.38 | Apba3,Mint3 | EGW03128 |  | 0.96 |  |  |
|  |  |  | 3.22 | Egr1,Egr-1,Krox-24 | EGW06690 |  | 0.96 |  |  |
|  |  |  | 3.01 | Aes | EGW03108 |  | 0.95 |  |  |
|  |  | -1.74 | -3.04 | Gnptab,Gnpta,Kiaa1208 | EGV93312 | 0.61 | 0.95 |  |  |
|  |  |  | 3.22 | Psmd9 | EGW07471 |  | 0.95 |  |  |
|  |  |  | -3.05 | Aff4 | EGW03017 |  | 0.95 |  |  |
|  |  | -1.91 | -3.18 | Ncoa3,Aib1,Pcip,Rac3,Tram1 | EGV97663 | 0.64 | 0.95 |  |  |
|  |  | 2.26 | 3.34 | Bhlhe40,Bhlhb2,Clast5,Stra13 | EGV92035 | 0.81 | 0.95 |  |  |
|  |  | -2.49 | -3.35 | Znf180 | EGW14754 | 0.69 | 0.95 |  |  |
|  |  | -3.11 | -3.52 | Ahi1 | EGV96104 | 0.71 | 0.95 |  |  |
|  |  | -1.83 | -3.10 | Tbl1xr1 | EGW10254 | 0.61 | 0.95 |  |  |
|  |  |  | -2.99 | Wrn | EGV98431 |  | 0.95 |  |  |
|  |  | -2.79 | -3.20 | Gtf2i | EGV96864 | 0.82 | 0.95 |  |  |
|  |  |  | 3.11 | Hmga1 | EGW01700 |  | 0.95 |  |  |
|  |  |  | -3.56 | Znf791 | EGV96159 |  | 0.95 |  |  |
|  |  |  | -3.29 | Cnot6 | EGW02876 |  | 0.95 |  |  |
|  |  |  | 3.01 | Znf593,Zfp593 | EGW10195 |  | 0.95 |  |  |
|  |  |  | -3.57 | Vgll3 | EGW00233 |  | 0.95 |  |  |
|  |  |  | -3.26 | Znf280c,Kiaa1584,Suhw3,Zfp280c | EGW03595 |  | 0.95 |  |  |
|  |  |  | -3.39 | Mier3,Qtsa-14674,Qtsa-16550 | EGV96933 |  | 0.95 |  |  |
|  |  | -2.15 | -3.29 | Znf770,Zfp770 | EGV92902 | 0.63 | 0.95 |  |  |
|  |  | 2.07 | 3.49 | Gata2 | EGW09469 | 0.73 | 0.95 |  |  |
|  |  | 2.49 | 3.29 | Znf385a,Hzf,Rzf,Znf385 | EGW09966 | 0.79 | 0.95 |  |  |
|  |  |  | -3.05 | Lrif1 | EGW03533 |  | 0.95 |  |  |
|  |  |  | -3.32 | Arid4a,Rbbp1,Rbp1 | EGW06494 |  | 0.95 |  |  |
|  |  |  | -3.96 | Foxi3 | EGW13915 |  | 0.95 |  |  |
|  |  |  | -3.56 | Ddx20,Dp103,Gemin3 | EGW10738 |  | 0.95 |  |  |
|  |  |  | -3.75 | Mybl1,Amyb | EGW04194 |  | 0.95 |  |  |
|  |  |  | 3.12 | Cbx4,Pc2 | EGW08244 |  | 0.95 |  |  |
|  |  |  | -2.99 | Kat2b,Pcaf | EGV96425 |  | 0.95 |  |  |
|  |  |  | -3.03 | Znf175 | EGW12542 |  | 0.95 |  |  |
|  |  |  | -3.08 | Znf43,Kox27,Znf39,Znf39l1 | EGV93056 |  | 0.95 |  |  |
|  |  |  | -3.26 | Znf426,Zfp426 | EGV96226 |  | 0.95 |  |  |
|  |  |  | 3.31 | Snai1,Sna | EGW05514 |  | 0.95 |  |  |
|  |  | -2.49 |  | Timeless | EGW12497 | 0.85 |  |  |  |
|  |  |  |  |  |  |  |  |  |  |
|  | **Differentiation** | 6.67 | 15.04 | Ccdc135 | EGW12725 | 0.89 | 1.00 |  |  |
|  |  |  | 6.11 | Gm16517 | EGV99459 |  | 0.99 |  |  |
|  |  | -2.99 | -4.54 | Ttc3,Dcrr1,Rnf105,Tprd | EGW12577 | 0.91 | 0.98 |  |  |
|  |  | -1.73 | -4.06 | Ubn1 | EGW05579 | 0.59 | 0.98 |  |  |
|  |  |  | 4.21 | Nbl1 | EGV96847 |  | 0.98 |  |  |
|  |  |  | -4.39 | Ift80 | EGW14558 |  | 0.98 |  |  |
|  |  |  | -5.23 | Krt80 | EGW13664 |  | 0.98 |  |  |
|  |  |  | 3.93 | Lgals1 | EGV94322 |  | 0.96 |  |  |
|  |  |  | 3.96 | Sbsn | EGW03711 |  | 0.96 |  |  |
|  |  |  | -6.07 | Tdrkh | EGW10750 |  | 0.96 |  |  |
|  |  |  | -3.53 | Strbp,Spnr | EGW04607 |  | 0.96 |  |  |
|  |  | -1.86 | -3.54 | Rod1 | EGW00664 | 0.61 | 0.96 |  |  |
|  |  |  | -3.15 | Tanc2 | EGV96769 |  | 0.95 |  |  |
|  |  |  | -3.15 | Lkap | EGV92997 |  | 0.95 |  |  |
|  |  |  | 3.08 | Klf4,Ezf,Gklf,Zie | EGV91809 |  | 0.95 |  |  |
|  |  | 3.86 |  | Dzip1,Kiaa0996 | EGV94434 | 0.88 |  |  |  |
|  |  |  |  |  |  |  |  |  |  |
|  | **Proliferation** |  | 5.74 | Pthlh,Pthrp | EGW08966 |  | 0.98 |  |  |
|  |  |  | -4.05 | Kiaa1524 | EGV96784 |  | 0.98 |  |  |
|  |  |  | 8.20 | Csf2,Csfgm | EGW08817 |  | 0.96 |  |  |
|  |  |  | -3.98 | Mll,All1,Hrx,Mll1 | EGW05718 |  | 0.96 |  |  |
|  |  |  | -3.23 | Tsc1 | EGW02657 |  | 0.95 |  |  |
|  |  | -2.31 |  | Cdca7 | EGV95681 | 0.83 |  |  |  |
|  |  |  |  |  |  |  |  |  |  |
|  | **Microtubules** |  | 6.72 | Map6 | EGW15319 |  | 0.99 |  |  |
|  |  |  | 4.16 | Map1s | EGV97464 |  | 0.98 |  |  |
|  |  | -2.78 | -4.09 | Kif14,Kiaa0042 | EGW02200 | 0.83 | 0.98 |  |  |
|  |  |  | -3.55 | Kif2a,Kif2 | EGV92062 |  | 0.96 |  |  |
|  |  |  | -3.84 | Dnahc8 | EGW02903 |  | 0.96 |  |  |
|  |  | 1.79 | 3.22 | Lmna | EGW07066 | 0.65 | 0.96 |  |  |
|  |  | -1.96 | -3.26 | Kif1c | EGV99747 | 0.62 | 0.95 |  |  |
|  |  | -2.24 | -3.13 | Kif20b,Mphosph1 | EGW00741 | 0.83 | 0.95 |  |  |
|  |  | -1.69 | -3.02 | Cenpe | EGW13494 | 0.60 | 0.95 |  |  |
|  |  | -2.52 |  | Kif4,Kif4a,Kns4 | EGV92321 | 0.80 |  |  |  |
|  |  | -3.00 |  | Mybl2,Bmyb | EGW09404 | 0.91 |  |  |  |
|  |  |  |  |  |  |  |  |  |  |
|  | **Actin cytoskeleton** |  | -11.81 | Dmd | EGW13991 |  | 0.99 |  |  |
|  |  |  | -5.83 | Dst | EGW14010 |  | 0.97 |  |  |
|  |  | 4.16 | 3.90 | Cdc42ep2,Borg1,Cep2 | EGW14746 | 0.88 | 0.96 |  |  |
|  |  |  | -3.39 | Nckap1 | EGV92867 |  | 0.96 |  |  |
|  |  |  | -3.32 | Fmnl2,Fhod2,Kiaa1902 | EGW13987 |  | 0.95 |  |  |
|  |  | 1.80 | 3.06 | Fhl3 | EGW00532 | 0.63 | 0.95 |  |  |
|  |  |  | -3.00 | Vcl | EGV97813 |  | 0.95 |  |  |
|  |  |  | -3.04 | Wasf1 | EGV98856 |  | 0.95 |  |  |
|  |  |  |  |  |  |  |  |  |  |
| **Protein synthesis** | **Ribosome** |  | 9.82 | Rpl36 | EGW04266 |  | 0.99 |  |  |
|  |  |  | 6.22 | Rpl36 | EGV98755 |  | 0.99 |  |  |
|  |  |  | 4.05 | Rpl36 | EGW06669 |  | 0.99 |  |  |
|  |  |  | 12.42 | Rpl18a | EGV94985 |  | 0.98 |  |  |
|  |  |  | 6.77 | Rpl18a | EGV98345 |  | 0.98 |  |  |
|  |  |  | 5.76 | Rpl18a | EGV91287 |  | 0.98 |  |  |
|  |  |  | 2.75 | Rpl18a | EGW09096 |  | 0.98 |  |  |
|  |  |  | 2.38 | Rpl18a | EGV97463 |  | 0.98 |  |  |
|  |  |  | 4.77 | Rps10 | EGW08573 |  | 0.98 |  |  |
|  |  |  | 2.59 | Rps10 | EGW01519 |  | 0.98 |  |  |
|  |  |  | 4.67 | Rps15,Rig | EGW03732 |  | 0.98 |  |  |
|  |  |  | 3.45 | Rps15,Rig | EGW04167 |  | 0.98 |  |  |
|  |  |  | 4.12 | Mrpl36 | EGW14938 |  | 0.98 |  |  |
|  |  |  | 4.35 | Mrpl43 | EGW13045 |  | 0.98 |  |  |
|  |  |  | 4.46 | Mrp63 | EGV92219 |  | 0.98 |  |  |
|  |  |  | 4.45 | Mrpl34 | EGV97487 |  | 0.98 |  |  |
|  |  |  | 6.52 | Rps16 | EGW02822 |  | 0.98 |  |  |
|  |  |  | 2.22 | Rps16 | EGV93048 |  | 0.98 |  |  |
|  |  |  | 4.78 | Plekhg6 | EGV98513 |  | 0.98 |  |  |
|  |  |  | 20.75 | Rps18 | EGV99016 |  | 0.96 |  |  |
|  |  |  | 4.26 | Rps18 | EGW04961 |  | 0.96 |  |  |
|  |  |  | 3.05 | Rps18 | EGW13313 |  | 0.96 |  |  |
|  |  | 2.14 | 3.57 | Pcbp4 | EGW08877 | 0.82 | 0.96 |  |  |
|  |  |  | 3.51 | Rps13 | EGW01891 |  | 0.96 |  |  |
|  |  |  | 3.47 | Rpl28 | EGW07502 |  | 0.96 |  |  |
|  |  |  | 3.47 | Rplp1,Rrp1 | EGW14924 |  | 0.96 |  |  |
|  |  |  | 2.74 | Rplp1,Rrp1 | EGV98916 |  | 0.96 |  |  |
|  |  |  | 2.65 | Rplp1,Rrp1 | EGW11932 |  | 0.96 |  |  |
|  |  |  | 2.34 | Rplp1,Rrp1 | EGV92231 |  | 0.96 |  |  |
|  |  |  | 3.15 | Eif6,Eif3a,Itgb4bp,Ok/Sw-Cl.27 | EGW02770 |  | 0.95 |  |  |
|  |  |  | 3.40 | Erbb3 | EGW12520 |  | 0.95 |  |  |
|  |  | 1.79 | 3.02 | Mrpl14 | EGW01349 | 0.61 | 0.95 |  |  |
|  |  |  | 3.32 | Rpl12 | EGV91715 |  | 0.95 |  |  |
|  |  |  | 3.18 | Rpl12 | EGW09574 |  | 0.95 |  |  |
|  |  |  | 3.46 | Rpl32 | EGV94768 |  | 0.95 |  |  |
|  |  |  | 3.22 | Rpl19 | EGV98458 |  | 0.95 |  |  |
|  |  |  | 2.45 | Rpl19 | EGW04931 |  | 0.95 |  |  |
|  |  |  | 2.42 | Rpl19 | EGW13056 |  | 0.95 |  |  |
|  |  |  | 2.23 | Rpl19 | EGW11358 |  | 0.95 |  |  |
|  |  |  | 2.01 | Rpl19 | EGV91367 |  | 0.95 |  |  |
|  |  |  | -3.12 | Mrpl15 | EGW09774 |  | 0.95 |  |  |
|  |  | 1.69 | 3.02 | Fth1,Fth | EGV98455 | 0.62 | 0.95 |  |  |
|  |  |  | -3.16 | Exosc9 | EGW01325 |  | 0.95 |  |  |
|  |  |  |  |  |  |  |  |  |  |
|  | **Translation** | -2.24 | -7.27 | Larp4b | EGV95107 | 0.78 | 1.00 |  |  |
|  |  |  | 4.39 | Mrps18a | EGW14044 |  | 0.98 |  |  |
|  |  |  | -7.25 | Eif2c3,Ago3 | EGW01096 |  | 0.96 |  |  |
|  |  |  | -3.87 | EGV93609 | EGV93609 |  | 0.96 |  |  |
|  |  | -2.24 | -3.51 | Ddx50 | EGV93790 | 0.83 | 0.96 |  |  |
|  |  |  | 3.50 | Rps8 | EGV98707 |  | 0.96 |  |  |
|  |  |  | -3.66 | Tnks,Parp5a,Parpl,Tin1,Tinf1 | EGV98450 |  | 0.96 |  |  |
|  |  | -2.44 | -2.86 | Eif5 | EGV94879 | 0.80 | 0.96 |  |  |
|  |  | -3.49 | -3.29 | Eif4a2 | EGW01984 | 0.92 | 0.96 |  |  |
|  |  | -2.15 | -3.20 | Dhx29 | EGV96945 | 0.76 | 0.95 |  |  |
|  |  | -2.17 | -2.72 | Larp4 | EGW13334 | 0.63 | 0.95 |  |  |
|  |  | -2.17 | -3.48 | Larp4 | EGV93615 | 0.63 | 0.95 |  |  |
|  |  |  | 3.38 | Sars2 | EGV93032 |  | 0.95 |  |  |
|  |  | -2.22 |  | Smg5,Est1b,Kiaa1089 | EGW09206 | 0.84 |  |  |  |
|  |  |  |  |  |  |  |  |  |  |
|  | **RNA processing** |  | 5.52 | Sf3b5 | EGW02527 |  | 0.99 |  |  |
|  |  |  | 4.49 | Imp3 | EGW03778 |  | 0.98 |  |  |
|  |  |  | 4.37 | Pop7,Rpp20 | EGW06330 |  | 0.98 |  |  |
|  |  | -2.15 | -4.04 | Fmr1 | EGW09643 | 0.71 | 0.98 |  |  |
|  |  |  | -3.20 | Rngtt,Cap1a | EGV92274 |  | 0.98 |  |  |
|  |  |  | -4.91 | Rngtt,Cap1a | EGV93811 |  | 0.98 |  |  |
|  |  |  | 5.02 | Ctu1 | EGW11580 |  | 0.98 |  |  |
|  |  |  | 6.16 | Mrm1 | EGV94922 |  | 0.97 |  |  |
|  |  |  | 4.77 | Mettl19 | EGV91596 |  | 0.97 |  |  |
|  |  |  | 3.89 | Rpusd3 | EGW06198 |  | 0.96 |  |  |
|  |  |  | 3.50 | Prpf31 | EGW07369 |  | 0.96 |  |  |
|  |  | -1.78 | -3.38 | Smek2,Kiaa1387,Pp4r3b,Ppp4r3b | EGW07856 | 0.63 | 0.96 |  |  |
|  |  |  | -3.32 | Cnot6l | EGW08961 |  | 0.96 |  |  |
|  |  |  | 3.23 | Tbl3 | EGW04439 |  | 0.95 |  |  |
|  |  |  | -3.24 | Prpf39 | EGW09175 |  | 0.95 |  |  |
|  |  |  | -3.36 | Prpf39 | EGW09176 |  | 0.95 |  |  |
|  |  |  | -3.25 | Papolg | EGW01801 |  | 0.95 |  |  |
|  |  |  | 3.32 | Zfp36,Tis11,Tis11a | EGV93044 |  | 0.95 |  |  |
|  |  |  | -3.21 | Upf2,Kiaa1408,Rent2 | EGV95423 |  | 0.95 |  |  |
|  |  |  | -3.46 | Upf2,Kiaa1408,Rent2 | EGV95423 |  | 0.95 |  |  |
|  |  |  | -3.30 | Utp14b,Jsd | EGW14732 |  | 0.95 |  |  |
|  |  |  | 3.03 | Mex3d,Rkhd1 | EGV99510 |  | 0.95 |  |  |
|  |  |  | -3.09 | Mtpap | EGV95239 |  | 0.95 |  |  |
|  |  |  | 3.23 | Trmu | EGW00758 |  | 0.95 |  |  |
|  |  |  | 3.89 | Adat3 | EGV99525 |  | 0.95 |  |  |
|  |  | -2.40 |  | Srsf6,Sfrs6,Srp55 | EGW07715 | 0.82 |  |  |  |
|  |  | -2.00 |  | Hnrnpc,Hnrpc | EGW08439 | 0.80 |  |  |  |
|  |  |  |  |  |  |  |  |  |  |
|  | **RE/Golgi** |  | 6.69 | Selm | EGW07118 |  | 0.99 |  |  |
|  |  |  | 4.44 | Sulf2 | EGV97661 |  | 0.98 |  |  |
|  |  |  | -4.18 | Gcc2 | EGW10288 |  | 0.98 |  |  |
|  |  | -2.41 | -4.39 | Acer3,Aphc,Phca | EGW04282 | 0.62 | 0.98 |  |  |
|  |  |  | 4.54 | Porcn | EGW06115 |  | 0.98 |  |  |
|  |  |  | -13.75 | Cyp4b1,Cyp4b-1 | EGV94559 |  | 0.98 |  |  |
|  |  |  | 4.56 | Xylt2 | EGW08286 |  | 0.97 |  |  |
|  |  |  | -5.25 | Fmo5 | EGW09227 |  | 0.97 |  |  |
|  |  |  | 4.81 | Fut1 | EGW03975 |  | 0.96 |  |  |
|  |  |  | 6.06 | Zfyve1,Dfcp1,Kiaa1589,Taff1,Znfn2a1 | EGW01617 |  | 0.96 |  |  |
|  |  | -1.73 | -3.91 | Ascc3,Helic1 | EGW08159 | 0.61 | 0.96 |  |  |
|  |  |  | -3.96 | Sacm1l | EGW08360 |  | 0.96 |  |  |
|  |  |  | -3.93 | Slc35a3 | EGW02964 |  | 0.96 |  |  |
|  |  |  | 3.47 | Rcn3 | EGV99178 |  | 0.96 |  |  |
|  |  |  | 3.39 | Inm02 | EGW07333 |  | 0.96 |  |  |
|  |  |  | -3.49 | Abca5 | EGW09269 |  | 0.96 |  |  |
|  |  |  | -3.41 | Ash1l | EGV98072 |  | 0.96 |  |  |
|  |  | -2.89 | -3.87 | Fktn | EGV97303 | 0.70 | 0.95 |  |  |
|  |  |  | -3.13 | Trip11 | EGW14619 |  | 0.95 |  |  |
|  |  |  | -3.00 | Ssr3 | EGV93553 |  | 0.95 |  |  |
|  |  | -1.91 | -3.15 | Sh3d19 | EGW04045 | 0.62 | 0.95 |  |  |
|  |  |  | 3.07 | Rcl | EGW09325 |  | 0.95 |  |  |
|  |  |  | 3.06 | Fv1 | EGW07325 |  | 0.95 |  |  |
|  |  |  | -3.90 | Fv1 | EGW01337 |  | 0.95 |  |  |
|  |  |  | -3.16 | Ubxn2a | EGV91882 |  | 0.95 |  |  |
|  |  |  | 3.35 | Dpm3 | EGV98090 |  | 0.95 |  |  |
|  |  |  | 3.66 | Chst14,D4st1 | EGW09319 |  | 0.95 |  |  |
|  |  |  | -3.38 | Pigw | EGV94927 |  | 0.95 |  |  |
|  |  | -2.72 | -3.08 | Nbea | EGW02378 | 0.64 | 0.95 |  |  |
|  |  | -2.72 | -3.94 | Nbea | EGW02379 | 0.64 | 0.95 |  |  |
|  |  | -2.08 | -3.41 | Nmt2 | EGV95455 | 0.62 | 0.95 |  |  |
|  |  | -2.08 | -3.02 | Nmt2 | EGV95456 | 0.62 | 0.95 |  |  |
|  |  |  | -2.99 | Cln5 | EGW02135 |  | 0.95 |  |  |
|  |  | 1.70 |  | Calr | EGW00614 | 0.62 |  |  |  |
|  |  | 2.06 |  | Herpud1 | EGW00061 | 0.81 |  |  |  |
|  |  |  |  |  |  |  |  |  |  |
|  | **chaperone** |  | -3.66 | Dnajc13,Kiaa0678,Rme8 | EGW05650 |  | 0.96 |  |  |
|  |  |  | -3.16 | Dnajc10,Erdj5,Jpdi | EGV92871 |  | 0.95 |  |  |
|  |  | -2.43 |  | Hsph1,Hsp105,Hsp110,Kiaa0201 | EGV93342 | 0.86 |  |  |  |
|  |  | -2.07 |  | Fkbp4,Fkbp52 | EGV98481 | 0.82 |  |  |  |
|  |  | -2.64 |  | Hsc70 | EGW10857 | 0.88 |  |  |  |
|  |  | -2.20 |  | Hspa8,Hsc70 | EGW10858 | 0.87 |  |  |  |
|  |  | -2.06 |  | Hspa8,Hsc70 | EGW10859 | 0.87 |  |  |  |
|  |  | -2.49 |  | Hspa8,Hsc70 | EGW07857 | 0.87 |  |  |  |
|  |  | -2.02 |  | Hspa8,Hsc70,Hsc73 | EGW02963 | 0.81 |  |  |  |
|  |  | -2.01 |  | Hspa8,Hsc70,Hsc73 | EGW07858 | 0.81 |  |  |  |
|  |  | -1.86 |  | Hspa8,Hsc70,Hsc73 | EGW08749 | 0.81 |  |  |  |
|  |  |  |  |  |  |  |  |  |  |
|  | **Protein modification** |  | 9.47 | Fkbpl | EGV95253 |  | 1.00 |  |  |
|  |  |  | -3.51 | Ate1,Qfla-16011 | EGW11696 |  | 0.96 |  |  |
|  |  |  | -3.39 | Slmap,Kiaa1601,Slap | EGW00679 |  | 0.96 |  |  |
|  |  |  | 3.29 | Fkbp11 | EGW10401 |  | 0.96 |  |  |
|  |  |  |  |  |  |  |  |  |  |
| **Protein degradation** | **Proteasome** | 1.81 | 4.21 | Psmb8 | EGW09109 | 0.64 | 0.98 |  |  |
|  |  |  | -3.79 | Spopl | EGW06666 |  | 0.96 |  |  |
|  |  | -2.09 | -3.51 | Casp8ap2 | EGV93826 | 0.63 | 0.96 |  |  |
|  |  |  | 3.03 | Psmg4 | EGV98213 |  | 0.95 |  |  |
|  |  |  |  |  |  |  |  |  |  |
|  | **Ubiquitination** |  | -4.16 | Wwp1 | EGW03234 |  | 0.98 |  |  |
|  |  |  | -4.37 | Dtx3l,Bbap | EGV96421 |  | 0.98 |  |  |
|  |  |  | -2.58 | Dcun1d1 | EGV93060 |  | 0.97 |  |  |
|  |  |  | -4.86 | Dcun1d1 | EGW10878 |  | 0.97 |  |  |
|  |  |  | -4.09 | Pcmtd1 | EGW04114 |  | 0.97 |  |  |
|  |  |  | -3.66 | Usp9x,Fafl,Fam | EGV96558 |  | 0.96 |  |  |
|  |  | -2.44 | -3.78 | Usp1 | EGW01028 | 0.81 | 0.96 |  |  |
|  |  | -2.44 | -4.83 | Usp1 | EGW01029 | 0.81 | 0.96 |  |  |
|  |  |  | -3.92 | Dzip3 | EGV96785 |  | 0.96 |  |  |
|  |  | -1.91 | -3.51 | Nedd4,Kiaa0093,Nedd4-1,Pig53 | EGV99884 | 0.68 | 0.96 |  |  |
|  |  |  | -3.33 | Huwe1,Kiaa0312,Kiaa1578,Ureb1 | EGW02329 |  | 0.96 |  |  |
|  |  | -2.73 | -3.33 | Usp31,Kiaa1203 | EGW13022 | 0.85 | 0.96 |  |  |
|  |  | -2.27 | -3.36 | G2e3,Kiaa1333 | EGV97534 | 0.77 | 0.96 |  |  |
|  |  |  | -3.35 | Ube4a | EGW05712 |  | 0.96 |  |  |
|  |  |  | -3.30 | Hace1,Kiaa1320 | EGW13180 |  | 0.96 |  |  |
|  |  |  | -3.15 | Usp24 | EGV91717 |  | 0.95 |  |  |
|  |  |  | -3.17 | Birc6,Kiaa1289 | EGW02704 |  | 0.95 |  |  |
|  |  |  | 3.18 | Mib2,Skd | EGW09937 |  | 0.95 |  |  |
|  |  |  | -3.27 | Usp37,Kiaa1594 | EGW11889 |  | 0.95 |  |  |
|  |  |  | -3.04 | Cul4b,Kiaa0695 | EGW01006 |  | 0.95 |  |  |
|  |  |  | -3.08 | Ube3a,E6ap,Epve6ap,Hpve6a | EGW13353 |  | 0.95 |  |  |
|  |  |  | -3.17 | Ptprh,Sap1 | EGW15098 |  | 0.95 |  |  |
|  |  |  | -3.07 | Fbxo30 | EGV99968 |  | 0.95 |  |  |
|  |  | -6.59 | -3.45 | Mkks,Bbs6 | EGV93075 | 0.80 | 0.95 |  |  |
|  |  |  | -3.43 | Usp12,Ubh1 | EGW00513 |  | 0.95 |  |  |
|  |  | 2.26 |  | Mdm2 | EGV96011 | 0.85 |  |  |  |
|  |  |  |  |  |  |  |  |  |  |
|  | **Proteolysis** | -4.72 | -5.59 | Senp7,Kiaa1707,Susp2 | EGW00792 | 0.82 | 0.99 |  |  |
|  |  |  | 7.90 | EGV96302 | EGV96302 |  | 0.97 |  |  |
|  |  |  | 8.90 | Gzmm | EGV99461 |  | 0.96 |  |  |
|  |  |  | -4.85 | Agbl3 | EGW11837 |  | 0.96 |  |  |
|  |  |  | 3.85 | Mmp9,Clg4b | EGV97682 |  | 0.96 |  |  |
|  |  |  | 3.58 | Ncln | EGW03112 |  | 0.96 |  |  |
|  |  | -1.85 | -3.57 | C2cd3 | EGW09027 | 0.61 | 0.96 |  |  |
|  |  | -2.88 | -3.42 | Ermp1,Fxna | EGW00424 | 0.87 | 0.96 |  |  |
|  |  |  | -3.47 | Prepl,Kiaa0436 | EGW03201 |  | 0.96 |  |  |
|  |  |  | -3.25 | Mll2,Alr,Kmt2b | EGW10393 |  | 0.95 |  |  |
|  |  | -1.74 | -3.19 | Ide | EGW00756 | 0.61 | 0.95 |  |  |
|  |  |  | 3.03 | Furin,Fur,Pcsk3 | EGW12796 |  | 0.95 |  |  |
|  |  |  | -3.03 | Senp6,Kiaa0797,Susp1 | EGW01187 |  | 0.95 |  |  |
|  |  | -2.31 | -3.17 | Senp5,Smt3ip3 | EGV96368 | 0.76 | 0.95 |  |  |
|  |  |  | -3.30 | Senp1,Supr2 | EGW14138 |  | 0.95 |  |  |
|  |  |  | -3.06 | Pppde1 | EGW00807 |  | 0.95 |  |  |
|  |  |  | -3.92 | Lonrf3,Rnf127 | EGV98376 |  | 0.95 |  |  |
|  |  |  | -3.05 | Phlpp2,Phlppl | EGW04131 |  | 0.95 |  |  |
|  |  |  | 3.22 | Adam19,Mltnb | EGW01403 |  | 0.95 |  |  |
|  |  |  | -2.98 | Lonrf1,Rnf191 | EGV96443 |  | 0.95 |  |  |
|  |  | -2.03 |  | Espl1,Esp1,Kiaa0165 | EGV95374 | 0.81 |  |  |  |
|  |  |  |  |  |  |  |  |  |  |
|  | **Lysosome** |  | -6.72 | Lyst | EGW00901 |  | 0.99 |  |  |
|  |  |  | -3.92 | Lmbrd1 | EGV92892 |  | 0.96 |  |  |
|  |  | 2.55 |  | Sidt2 | EGW10871 | 0.87 |  |  |  |
|  |  |  |  |  |  |  |  |  |  |
|  | **Peptidase** | 2.78 | 4.10 | Col7a1 | EGV92133 | 0.87 | 0.98 |  |  |
|  |  | 2.80 | 4.76 | Wfikkn1,Gasp2 | EGW04373 | 0.76 | 0.98 |  |  |
|  |  | 3.35 | 3.82 | Ctla2b | EGW06748 | 0.91 | 0.96 |  |  |
|  |  |  | -3.97 | Lnpep | EGV98387 |  | 0.96 |  |  |
|  |  |  | 11.33 | Rhbdl2 | EGV92571 |  | 0.95 |  |  |
|  |  |  |  |  |  |  |  |  |  |
| **Transport and secretion of protein** | **Endocytosis** |  | -4.45 | Pik3c2a,Cpk | EGV92624 |  | 0.98 |  |  |
|  |  |  | 4.18 | Vps37d | EGW01821 |  | 0.97 |  |  |
|  |  | -2.81 | -3.59 | Eea1 | EGW14167 | 0.79 | 0.96 |  |  |
|  |  |  | -3.13 | Picalm,Calm,Fit1 | EGW05901 |  | 0.95 |  |  |
|  |  |  | 3.47 | Ccdc115 | EGW14005 |  | 0.95 |  |  |
|  |  |  | -3.24 | Kiaa1033 | EGV93842 |  | 0.95 |  |  |
|  |  | 2.58 |  | Tmbim1,Recs1,Pp1201,Psec0158 | EGW09735 | 0.86 |  |  |  |
|  |  |  |  |  |  |  |  |  |  |
|  | **Exocitosis** |  | 5.48 | S100a6,Cacy | EGV98153 |  | 0.99 |  |  |
|  |  | -2.54 | -4.70 | Mum1l1 | EGW13755 | 0.66 | 0.98 |  |  |
|  |  |  | -4.30 | Ptx3 | EGV93547 |  | 0.96 |  |  |
|  |  |  | 3.53 | S100a5,S100d | EGV98152 |  | 0.95 |  |  |
|  |  | -3.32 |  | Cpne1,Cpn1 | EGW02780 | 0.90 |  |  |  |
|  |  |  |  |  |  |  |  |  |  |
|  | **Organization organelles** |  | -6.85 | Dync2h1,Dhc1b,Dlp4,Dnch2,Dnchc2 | EGV95528 |  | 0.99 |  |  |
|  |  |  | -4.96 | Ppp2r5a | EGV96684 |  | 0.96 |  |  |
|  |  |  | -3.31 | Hps3 | EGW13340 |  | 0.95 |  |  |
|  |  |  | -3.15 | Lztfl1 | EGW08366 |  | 0.95 |  |  |
|  |  |  |  |  |  |  |  |  |  |
|  | **Protein secretion** |  | 6.04 | Olfm2 | EGV96221 |  | 0.98 |  |  |
|  |  |  | 4.52 | R102.4 | EGV97886 |  | 0.96 |  |  |
|  |  |  |  |  |  |  |  |  |  |
|  | **Transport protein** | -1.97 | -6.04 | Vps13a,Chac,Kiaa0986 | EGW07549 | 0.66 | 0.99 |  |  |
|  |  |  | 8.94 | Ier5 | EGW10142 |  | 0.99 |  |  |
|  |  |  | 4.72 | Trappc5 | EGW00206 |  | 0.98 |  |  |
|  |  |  | -4.49 | Chml | EGW05761 |  | 0.98 |  |  |
|  |  |  | 4.14 | Arl4c,Arl7 | EGW11166 |  | 0.98 |  |  |
|  |  |  | -4.65 | Wdr19,Kiaa1638 | EGW12074 |  | 0.98 |  |  |
|  |  |  | -4.18 | Rhoq,Tc10 | EGW14329 |  | 0.98 |  |  |
|  |  |  | 5.81 | Arl11 | EGW04616 |  | 0.98 |  |  |
|  |  |  | 4.38 | Slc7a6os | EGW10091 |  | 0.97 |  |  |
|  |  |  | 3.62 | Rap2b | EGW05407 |  | 0.96 |  |  |
|  |  | -2.22 | -2.99 | Ipo7,Ranbp7 | EGW10582 | 0.84 | 0.95 |  |  |
|  |  | -2.69 | -3.15 | Kpna4,Qip1 | EGW02042 | 0.86 | 0.95 |  |  |
|  |  |  | -4.52 | Kpna6,Ipoa7 | EGW08341 |  | 0.95 |  |  |
|  |  |  | -3.07 | Lrrk2 | EGW00676 |  | 0.95 |  |  |
|  |  |  | 3.22 | Rhob,Arhb | EGW08219 |  | 0.95 |  |  |
|  |  |  | -3.48 | Rab11fip2,Kiaa0941 | EGV96719 |  | 0.95 |  |  |
|  |  | -2.80 |  | Kpnb1,Impnb | EGW05268 | 0.89 |  |  |  |
|  |  |  |  |  |  |  |  |  |  |
|  | **Transport** | -1.77 | -4.98 | Ranbp2 | EGW10295 | 0.63 | 0.99 |  |  |
|  |  |  | 10.40 | Kcnk15 | EGV98288 |  | 0.98 |  |  |
|  |  |  | 4.77 | EGW03121 | EGW01935 |  | 0.98 |  |  |
|  |  | 1.79 | 3.58 | Slc39a7,H2-Ke4,Hke4 | EGW09099 |  | 0.96 |  |  |
|  |  | 2.17 | 3.45 | Ano1 | EGW01935 | 0.65 | 0.96 |  |  |
|  |  | -2.42 | -3.06 | Slc39a10,Kiaa1265,Zip10 | EGV95466 | 0.76 | 0.95 |  |  |
|  |  |  |  |  |  |  |  |  |  |
|  | **Vesicles** |  | 4.57 | Cno | EGV91610 |  | 0.98 |  |  |
|  |  |  | -4.12 | Fcho2 | EGW04164 |  | 0.98 |  |  |
|  |  |  | 4.04 | C1qtnf5 | EGV96912 |  | 0.98 |  |  |
|  |  |  | -3.82 | Myo6,Kiaa0389 | EGW01186 |  | 0.96 |  |  |
|  |  | 2.22 |  | Zfpl1 | EGW12253 | 0.81 |  |  |  |
|  |  |  |  |  |  |  |  |  |  |
|  | **Vacuoles** |  | 3.97 | Atp6v1g2,Atp6g2,Ng38 | EGV95297 |  | 0.95 |  |  |
|  |  |  | -3.28 | Ap1s2,Dc22 | EGW05846 |  | 0.95 |  |  |
|  |  |  |  |  |  |  |  |  |  |
|  | **Protein binding** | -2.14 | -5.46 | Trim59,Mrf1 | EGW14563 | 0.59 | 0.99 |  |  |
|  |  | -3.03 | -4.10 | Brca1 | EGW08077 | 0.90 | 0.98 |  |  |
|  |  |  | 4.83 | Hic1 | EGW14223 |  | 0.98 |  |  |
|  |  |  | -4.01 | Zbtb41 | EGW10913 |  | 0.98 |  |  |
|  |  |  | -3.42 | Mid2,Fxy2,Trim1 | EGW13074 |  | 0.95 |  |  |
|  |  |  | -3.62 | Zbtb33,Kaiso | EGW01011 |  | 0.95 |  |  |
|  |  |  |  |  |  |  |  |  |  |
|  | **Cytoskeleton** | -2.03 | -5.33 | Cep290 | EGV96278 | 0.64 | 0.99 |  |  |
|  |  | 1.92 | 5.11 | Dusp6,Mkp3 | EGV92485 | 0.62 | 0.99 |  |  |
|  |  | 1.79 | 4.60 | Tubb6 | EGW00717 | 0.64 | 0.98 |  |  |
|  |  | 1.92 | 3.47 | Tubb6 | EGV96313 | 0.64 | 0.98 |  |  |
|  |  | 2.88 | 4.05 | Frmd8 | EGW14742 | 0.89 | 0.98 |  |  |
|  |  | -2.11 | -4.12 | Ptpn4 | EGV99078 | 0.62 | 0.98 |  |  |
|  |  |  | -4.91 | Sfi1 | EGW07104 |  | 0.98 |  |  |
|  |  |  | 3.81 | Baiap2 | EGW06229 |  | 0.96 |  |  |
|  |  |  | -3.51 | Kifap3 | EGW10332 |  | 0.96 |  |  |
|  |  |  | -3.46 | Jak2 | EGW11816 |  | 0.96 |  |  |
|  |  |  | -3.14 | Cntln | EGW03864 |  | 0.95 |  |  |
|  |  |  | -3.47 | Cntln | EGW03865 |  | 0.95 |  |  |
|  |  |  | -4.46 | Cntln | EGW03522 |  | 0.95 |  |  |
|  |  |  | -2.99 | Camsap1l1 | EGW02198 |  | 0.95 |  |  |
|  |  |  | 3.21 | Mzt2 | EGW14250 |  | 0.95 |  |  |
|  |  |  | 3.59 | Fam110a | EGW06856 |  | 0.95 |  |  |
|  |  |  | 3.96 | Dusp5,Vh3 | EGW12856 |  | 0.95 |  |  |
|  |  |  | 3.04 | Dusp15 | EGW00290 |  | 0.95 |  |  |
|  |  |  | -4.90 | Pclo | EGW10218 |  | 0.95 |  |  |
|  |  |  |  |  |  |  |  |  |  |
| **Energy metabolism** | **ATP binding** | -5.72 | -8.29 | Atp11c | EGV96455 | 0.76 | 1.00 |  |  |
|  |  | -2.75 | -4.70 | Atad5,Frag1 | EGV94362 | 0.71 | 0.98 |  |  |
|  |  |  | -4.25 | Stard9 | EGV97229 |  | 0.98 |  |  |
|  |  | -2.97 | -4.41 | Atp7b,Wnd | EGV95005 | 0.62 | 0.98 |  |  |
|  |  |  | 3.89 | Atp5d | EGV99489 |  | 0.96 |  |  |
|  |  | -2.06 | -3.61 | Crybg3 | EGW10275 | 0.79 | 0.96 |  |  |
|  |  |  | -3.67 | Slfn12 | EGW14914 |  | 0.96 |  |  |
|  |  |  | 3.28 | Atp5i | EGW07978 |  | 0.96 |  |  |
|  |  |  | -3.31 | Atad2b,Kiaa1240 | EGV91883 |  | 0.95 |  |  |
|  |  |  |  |  |  |  |  |  |  |
|  | **Mitochondria** |  | 7.09 | Bola1 | EGV94781 |  | 1.00 |  |  |
|  |  |  | 4.14 | Isoc2a,Isoc2 | EGW07506 |  | 0.98 |  |  |
|  |  |  | 3.77 | Fxc1,Tim9b,Timm9b | EGW13693 |  | 0.96 |  |  |
|  |  |  | 3.98 | Mrpl53 | EGW11797 |  | 0.96 |  |  |
|  |  |  | 3.44 | Atp5e | EGV91885 |  | 0.96 |  |  |
|  |  |  | 3.77 | Dnlz | EGW12770 |  | 0.96 |  |  |
|  |  |  | 3.46 | Timm13,Tim13a,Timm13a | EGW01991 |  | 0.96 |  |  |
|  |  |  | 3.00 | Timm13,Tim13a,Timm13a | EGV99546 |  | 0.96 |  |  |
|  |  |  | 3.14 | Uqcr11,Uqcr | EGV99514 |  | 0.95 |  |  |
|  |  |  | 3.27 | Nme3 | EGW04425 |  | 0.95 |  |  |
|  |  |  |  |  |  |  |  |  |  |
|  | **NADH** |  | 4.44 | Ndufb7 | EGW00659 |  | 0.98 |  |  |
|  |  |  | -5.15 | Parp8 | EGV96795 |  | 0.98 |  |  |
|  |  |  | 4.23 | Cbr3 | EGW12588 |  | 0.98 |  |  |
|  |  |  | 4.59 | Cbr2 | EGW06280 |  | 0.97 |  |  |
|  |  |  | 3.10 | Ndufs7 | EGV99498 |  | 0.95 |  |  |
|  |  |  |  |  |  |  |  |  |  |
|  | **Redox** |  | -5.98 | Aox1,Ao,Ro | EGW11977 |  | 0.99 |  |  |
|  |  |  | -6.28 | Aox1,Ao,Ro | EGW11977 |  | 0.99 |  |  |
|  |  |  | -7.12 | Aox1 | EGV99451 |  | 0.99 |  |  |
|  |  |  | -6.06 | Pipox,Pso | EGW14985 |  | 0.96 |  |  |
|  |  | -1.80 | -3.82 | Plod2 | EGW10258 | 0.62 | 0.96 |  |  |
|  |  | 1.84 | 3.18 | Sh3bgrl3,P1725 | EGW10190 | 0.65 | 0.95 |  |  |
|  |  |  |  |  |  |  |  |  |  |
| **Metabolism** | **Biosynthesis** |  | 6.36 | Rsad1 | EGW08280 |  | 0.99 |  |  |
|  |  | 1.73 | 4.05 | Aprt | EGV94074 | 0.63 | 0.98 |  |  |
|  |  |  | 4.45 | Hsd3b2 | EGW09208 |  | 0.97 |  |  |
|  |  | 2.03 | 3.42 | Pmm1 | EGW14817 | 0.74 | 0.96 |  |  |
|  |  | 2.03 | 2.56 | Pmm1 | EGW13957 | 0.74 | 0.96 |  |  |
|  |  | -2.08 | -3.10 | Mtr | EGW01906 | 0.79 | 0.95 |  |  |
|  |  |  | -3.34 | Bco2 | EGW03823 |  | 0.95 |  |  |
|  |  | 3.49 |  | Tk1 | EGV97892 | 0.87 |  |  |  |
|  |  | -2.12 |  | Umps | EGV96405 | 0.80 |  |  |  |
|  |  | 2.03 |  | Fdft1,Erg9 | EGW10357 | 0.81 |  |  |  |
|  |  | -2.07 |  | Mthfd2,Nmdmc | EGW09962 | 0.82 |  |  |  |
|  |  | -2.19 |  | Nampt,Pbef1 | EGW07127 | 0.80 |  |  |  |
|  |  | -2.09 |  | Lct,Lph | EGW00948 | 0.81 |  |  |  |
|  |  | -2.72 |  | Pfas,Kiaa0361 | EGV93868 | 0.87 |  |  |  |
|  |  |  |  |  |  |  |  |  |  |
|  | **Catalysis** |  | -7.65 | Ppm1l,Pp2ce | EGW02040 |  | 0.98 |  |  |
|  |  | 2.51 | 5.66 | Spata20 | EGW08279 | 0.61 | 0.98 |  |  |
|  |  | 3.27 | 3.74 | Ppcdc,Coac | EGW04011 | 0.82 | 0.96 |  |  |
|  |  |  |  |  |  |  |  |  |  |
|  | **Lipid** |  | -5.09 | Idi1 | EGV95102 |  | 0.99 |  |  |
|  |  | 1.83 | 5.03 | Pafah1b3,Pafahg | EGW10452 | 0.63 | 0.99 |  |  |
|  |  | -2.29 | -4.61 | Sacs,Kiaa0730 | EGW04618 | 0.78 | 0.98 |  |  |
|  |  |  | -4.11 | Tecta | EGW10638 |  | 0.98 |  |  |
|  |  |  | -4.12 | Hsd17b7 | EGW06167 |  | 0.98 |  |  |
|  |  |  | 4.14 | Gpr39 | EGW02382 |  | 0.97 |  |  |
|  |  |  | -4.59 | Slc27a6,Acsvl2,Facvl2,Fatp1 | EGW06764 |  | 0.97 |  |  |
|  |  | -2.25 | -3.97 | Ankrd26 | EGW08323 | 0.73 | 0.96 |  |  |
|  |  | -3.26 | -3.97 | Prkar2b | EGW04272 | 0.82 | 0.96 |  |  |
|  |  |  | -3.28 | Ddhd2,Kiaa0725,Samwd1 | EGV94663 |  | 0.96 |  |  |
|  |  |  | -2.99 | Far1,Mlstd2 | EGW14513 |  | 0.95 |  |  |
|  |  |  | -3.47 | Etnk1 | EGV97870 |  | 0.95 |  |  |
|  |  |  | -3.04 | Osbpl8,Kiaa1451,Orp8,Osbp10 | EGV97330 |  | 0.95 |  |  |
|  |  |  | -3.11 | Acad11 | EGW05647 |  | 0.95 |  |  |
|  |  |  | 3.32 | Acaa1b,Acaa1 | EGW00823 |  | 0.95 |  |  |
|  |  |  | 2.24 | Acaa1b,Acaa1 | EGW00828 |  | 0.95 |  |  |
|  |  |  | -5.53 | Gpr116,Gprhep | EGW10645 |  | 0.95 |  |  |
|  |  | 2.24 |  | Pnpla2,Atgl | EGW05549 | 0.83 |  |  |  |
|  |  |  |  |  |  |  |  |  |  |
|  | **Carbohydrate metabolism** | 19.66 | 5.65 | Ldhc,Ldh-3,Ldh3 | EGW00383 | 0.98 | 0.99 |  |  |
|  |  | 1.95 | 4.86 | Dcxr | EGW06279 | 0.64 | 0.98 |  |  |
|  |  |  | 4.01 | Amdhd2 | EGW09758 |  | 0.98 |  |  |
|  |  |  | -4.11 | Gbe1 | EGW10062 |  | 0.98 |  |  |
|  |  |  | 6.50 | Nudt11,Dipp3b,Mncb-1696 | EGW11287 |  | 0.98 |  |  |
|  |  |  | 3.95 | Pgp | EGW04455 |  | 0.96 |  |  |
|  |  |  | 3.78 | Neu2 | EGV94738 |  | 0.96 |  |  |
|  |  |  | 3.58 | Galk1,Galk,Glk | EGW04660 |  | 0.96 |  |  |
|  |  | 2.03 | 3.15 | Ldha,Pig19 | EGW00382 | 0.82 | 0.95 |  |  |
|  |  |  | -3.20 | Ppp1cb,Tegg049h05.1 | EGV98330 |  | 0.95 |  |  |
|  |  |  | -3.11 | Ppip5k1,Hisppd2a,Kiaa0377,Vip1 | EGV97210 |  | 0.95 |  |  |
|  |  | 2.33 |  | Grina,Nmdara1 | EGV92726 | 0.85 |  |  |  |
|  |  |  |  |  |  |  |  |  |  |
| **Stress response** | **Cold chock** | 5.81 | 13.55 | Rbm3,Rnpl | EGW06112 | 0.96 | 1.00 |  |  |
|  |  | 3.32 | 7.77 | Cirbp,Cirp | EGV99492 | 0.90 | 1.00 |  |  |
|  |  |  |  |  |  |  |  |  |  |
|  | **Ras** |  | -3.28 | Shoc2 | EGW05944 |  | 0.95 |  |  |
|  |  |  |  |  |  |  |  |  |  |
|  | **Kinase** | 2.90 | 6.38 | Tk1 | EGV97892 | 0.87 | 0.99 |  |  |
|  |  | 2.90 | 4.56 | Tk1 | EGV92968 | 0.87 | 0.99 |  |  |
|  |  |  | -5.31 | Prkdc,Xrcc7 | EGW06573 |  | 0.99 |  |  |
|  |  |  | -4.36 | Mobkl1a | EGW00580 |  | 0.98 |  |  |
|  |  |  | -3.63 | Wnk1 | EGW09383 |  | 0.96 |  |  |
|  |  | -2.14 | -3.19 | Uhmk1,Kis,Kist | EGW06173 | 0.70 | 0.95 |  |  |
|  |  | -2.07 |  | Wnk2,Kiaa1760 | EGV99811 | 0.82 |  |  |  |
|  |  |  |  |  |  |  |  |  |  |
|  | **Methylation** |  | 3.15 | Comtd1 | EGV97825 |  | 0.95 |  |  |
|  |  |  |  |  |  |  |  |  |  |
|  | **P53** |  | 4.31 | Mif | EGW12157 |  | 0.98 |  |  |
|  |  | -1.74 | -3.11 | Ptges3 | EGW12489 | 0.62 | 0.95 |  |  |
|  |  |  |  |  |  |  |  |  |  |
|  | **MAPK** |  | -7.48 | Itga1 | EGV92178 |  | 0.99 |  |  |
|  |  |  | 3.93 | Gadd45b | EGW03148 |  | 0.96 |  |  |
|  |  |  | 3.27 | Mpg,Mid1 | EGW10378 |  | 0.95 |  |  |
|  |  |  |  |  |  |  |  |  |  |
|  | **ROX** |  | 3.05 | Romo1 | EGW02783 |  | 0.95 |  |  |
|  |  |  |  |  |  |  |  |  |  |
|  | **Signaling** | -2.27 | -7.25 | Rock1 | EGV91660 | 0.72 | 1.00 |  |  |
|  |  | -2.68 | -6.66 | Arhgap5,Rhogap5 | EGW12688 | 0.87 | 0.99 |  |  |
|  |  |  | 7.07 | Spry2 | EGV97540 |  | 0.99 |  |  |
|  |  | 2.22 | 5.97 | Cxcr7,Cmkor1,Rdc1 | EGW14644 | 0.79 | 0.99 |  |  |
|  |  | -2.82 | -6.29 | Cnksr2 | EGV98780 | 0.60 | 0.99 |  |  |
|  |  | -2.82 | -3.65 | Cnksr2 | EGV98779 | 0.60 | 0.99 |  |  |
|  |  | -2.46 | -5.20 | Myo9a,Myr7 | EGW04529 | 0.81 | 0.99 |  |  |
|  |  |  | 12.77 | Ngf,Ngfb | EGW00029 |  | 0.99 |  |  |
|  |  |  | -5.02 | Rpe65 | EGV93975 |  | 0.99 |  |  |
|  |  | -2.08 | -4.80 | Ralgapa1,Garnl1,Kiaa0884,Tulip1 | EGW11724 | 0.79 | 0.98 |  |  |
|  |  | -2.48 | -5.26 | Ppm1e,Camkn,Kiaa1072 | EGV96623 | 0.60 | 0.98 |  |  |
|  |  | -2.13 | -4.67 | Tbc1d8b | EGW07632 | 0.69 | 0.98 |  |  |
|  |  | -1.91 | -4.27 | Dennd4a | EGW10969 | 0.63 | 0.98 |  |  |
|  |  |  | -4.37 | Ccdc88a | EGW07854 |  | 0.98 |  |  |
|  |  |  | -4.36 | Akap11 | EGW09123 |  | 0.98 |  |  |
|  |  |  | -5.81 | Fam13b,Fam13b1 | EGW06699 |  | 0.98 |  |  |
|  |  |  | -4.51 | Tbck | EGW11325 |  | 0.98 |  |  |
|  |  | 2.51 | 4.37 | Il1rl1 | EGV92291 | 0.70 | 0.98 |  |  |
|  |  |  | -5.35 | Dennd5b | EGW10829 |  | 0.98 |  |  |
|  |  |  | 4.33 | Or5ak2 | EGW01526 |  | 0.98 |  |  |
|  |  |  | 4.80 | Gpr39 | EGW02382 |  | 0.97 |  |  |
|  |  | 2.11 | 5.21 | Camk2n2 | EGV97790 | 0.59 | 0.97 |  |  |
|  |  |  | -5.00 | Dnah12,Dnah12l,Dnah7l,Dnahc12 | EGW00688 |  | 0.96 |  |  |
|  |  |  | -4.03 | Olfr181,Mor184-4 | EGW12958 |  | 0.96 |  |  |
|  |  | -2.20 | -3.70 | Ralgapa2,Kiaa1272 | EGV95387 | 0.79 | 0.96 |  |  |
|  |  |  | -3.78 | Rasa2 | EGW00483 |  | 0.96 |  |  |
|  |  |  | -3.57 | Dennd4c | EGW14455 |  | 0.96 |  |  |
|  |  |  | 3.60 | Sphk1 | EGW04691 |  | 0.96 |  |  |
|  |  |  | 3.57 | Ddit4,Dig2,Redd1,Rtp801 | EGV98210 |  | 0.96 |  |  |
|  |  |  | -3.80 | Nlrx1 | EGV99100 |  | 0.96 |  |  |
|  |  | -2.07 | -3.68 | Arhgap19 | EGV99279 | 0.65 | 0.96 |  |  |
|  |  | -1.91 | -3.17 | Zfp106,H3a,Sh3bp3,Sirm,Znf474 | EGV97233 | 0.68 | 0.95 |  |  |
|  |  |  | -3.16 | Itsn2,Ese2,Sh3d1b | EGV95823 |  | 0.95 |  |  |
|  |  | -1.67 | -3.08 | Rock2 | EGW08488 | 0.60 | 0.95 |  |  |
|  |  | 2.37 | 3.16 | Dgka,Dagk1 | EGW12525 | 0.84 | 0.95 |  |  |
|  |  | -2.07 | -3.00 | Ect2 | EGW00004 | 0.78 | 0.95 |  |  |
|  |  |  | -3.11 | Nf1 | EGV94354 |  | 0.95 |  |  |
|  |  | -2.19 | -3.38 | Snx27 | EGW03080 | 0.70 | 0.95 |  |  |
|  |  | -1.91 | -3.10 | Iqgap2 | EGW04240 | 0.63 | 0.95 |  |  |
|  |  | -1.81 | -2.99 | Slc6a15,B0at2,Ntt73 | EGW08102 | 0.59 | 0.95 |  |  |
|  |  |  | -3.37 | Olr1496 | EGW12338 |  | 0.95 |  |  |
|  |  | -2.25 | -2.99 | Snx25 | EGW15040 | 0.77 | 0.95 |  |  |
|  |  |  | -3.04 | Arhgap12 | EGW05184 |  | 0.95 |  |  |
|  |  | 3.25 | 4.52 | Lat | EGV95597 | 0.59 | 0.95 |  |  |
|  |  |  | -3.00 | Tlr4 | EGW02588 |  | 0.95 |  |  |
|  |  | -2.85 | -3.05 | Aim2,Gm1313,Ifi210 | EGV93426 | 0.75 | 0.95 |  |  |
|  |  |  | -3.30 | Rpgrip1l | EGV92009 |  | 0.95 |  |  |
|  |  |  | -3.02 | Rabgap1l | EGV93294 |  | 0.95 |  |  |
|  |  |  | -3.02 | Tbc1d12,Kiaa0608 | EGV98687 |  | 0.95 |  |  |
|  |  |  | 3.67 | Socs1,Cish1,Ssi1 | EGV96317 |  | 0.95 |  |  |
|  |  |  | 3.16 | Nfkbib | EGV93028 |  | 0.95 |  |  |
|  |  | -5.36 |  | Cxcl3,Cinc2 | EGW00999 | 0.82 |  |  |  |
|  |  | -2.04 |  | Rangap1,Fug1 | EGW05207 | 0.81 |  |  |  |
|  |  | -3.09 |  | Pask,Kiaa0135 | EGV95324 | 0.81 |  |  |  |
|  |  | 2.71 |  | Npr2 | EGW07876 | 0.85 |  |  |  |
|  |  |  |  |  |  |  |  |  |  |
|  | **kinase signaling** | -2.10 | -4.25 | Nek1 | EGW01379 | 0.71 | 0.98 |  |  |
|  |  |  | -4.50 | Peak1,Kiaa2002,Sgk269 | EGW03772 |  | 0.98 |  |  |
|  |  |  | -4.33 | Pak3 | EGW12031 |  | 0.98 |  |  |
|  |  |  | -4.15 | Csnk1g3 | EGV94535 |  | 0.97 |  |  |
|  |  | -2.21 | -3.90 | Cdc42bpa,Kiaa0451 | EGW11188 | 0.71 | 0.96 |  |  |
|  |  |  | -3.41 | Tlk1 | EGV95663 |  | 0.96 |  |  |
|  |  |  | -3.39 | Nek7 | EGW10922 |  | 0.96 |  |  |
|  |  | -2.95 | -3.49 | Mlkl | EGW07301 | 0.79 | 0.96 |  |  |
|  |  | -2.17 | -3.11 | Plk4,Sak | EGW06341 | 0.80 | 0.95 |  |  |
|  |  |  | 3.79 | Dyrk3 | EGV92824 |  | 0.95 |  |  |
|  |  |  | 3.20 | Sgk223,D8ertd82e | EGV96442 |  | 0.95 |  |  |
|  |  |  | -3.30 | Sik2,Snf1lk2 | EGW03804 |  | 0.95 |  |  |
|  |  |  | -3.04 | Mastl,Gw,Gwl | EGV97839 |  | 0.95 |  |  |
|  |  | -2.24 | -3.10 | Hipk3,Fist3 | EGV94193 | 0.74 | 0.95 |  |  |
|  |  |  | -3.81 | Ttbk2,Kiaa0847,Ttbk1 | EGV97224 |  | 0.95 |  |  |
|  |  |  |  |  |  |  |  |  |  |
|  | **G protein signaling** |  | -3.99 | Dock11,Ziz2 | EGV98377 |  | 0.98 |  |  |
|  |  |  | -4.27 | Gnaq | EGW06793 |  | 0.97 |  |  |
|  |  |  | -5.13 | Gnaq | EGW10015 |  | 0.97 |  |  |
|  |  |  | 3.06 | Gnat1 | EGW08913 |  | 0.95 |  |  |
|  |  |  |  |  |  |  |  |  |  |
|  | **Wnt signaling** | -2.07 | -4.17 | Ppp2r3a | EGV96709 | 0.63 | 0.98 |  |  |
|  |  |  | -3.28 | Mllt3 | EGV95883 |  | 0.95 |  |  |
|  |  |  | -3.63 | Rnf138 | EGW05845 |  | 0.95 |  |  |
|  |  |  |  |  |  |  |  |  |  |
| **Cell death** | **Apoptosis** |  | 23.05 | Il33 | EGW13913 |  | 1.00 |  |  |
|  |  | 2.68 | 6.22 | Aen,Isg20l1 | EGW13741 | 0.86 | 0.99 |  |  |
|  |  | 2.02 | 6.58 | Phlda3 | EGW02186 | 0.75 | 0.99 |  |  |
|  |  |  | 5.58 | Tnfrsf12a | EGV93656 |  | 0.99 |  |  |
|  |  |  | 5.94 | Fhl2 | EGV98814 |  | 0.99 |  |  |
|  |  |  | -4.44 | Phip | EGV98200 |  | 0.98 |  |  |
|  |  |  | 4.44 | Zc3h12a | EGW00553 |  | 0.98 |  |  |
|  |  |  | -4.56 | Ifih1 | EGW01901 |  | 0.98 |  |  |
|  |  | 1.77 | 3.90 | Cyr61,Ccn1,Igfbp10 | EGV99691 | 0.62 | 0.96 |  |  |
|  |  |  | -3.34 | Opt | EGV93284 |  | 0.96 |  |  |
|  |  | 1.96 | 3.18 | Bax | EGW03981 | 0.66 | 0.95 |  |  |
|  |  |  | 3.31 | Cidec,Fsp27 | EGW06200 |  | 0.95 |  |  |
|  |  | -3.22 | -3.27 | Ppid | EGW00520 | 0.78 | 0.95 |  |  |
|  |  |  | -3.09 | Fnip1,Kiaa1961 | EGW08818 |  | 0.95 |  |  |
|  |  |  | 5.32 | Fam176a | EGV96803 |  | 0.95 |  |  |
|  |  |  | 3.31 | Nle1 | EGV94393 |  | 0.95 |  |  |
|  |  | 2.07 |  | Lrp1,A2mr | EGW02265 | 0.82 |  |  |  |
|  |  | -2.34 |  | Bub1b,Mad3l | EGW14538 | 0.81 |  |  |  |
|  |  | 2.31 |  | Pdcd4,Ma3,Tis | EGW05942 | 0.80 |  |  |  |
|  |  |  |  |  |  |  |  |  |  |
|  | **Autophagy** |  | -6.42 | Atg4c | EGW01026 |  | 0.98 |  |  |
|  |  |  | -3.87 | Rb1cc1 | EGW10581 |  | 0.96 |  |  |
|  |  | 5.56 |  | Tp53inp2,Dor,Trp53inp2 | EGW02761 | 0.83 |  |  |  |
|  |  |  |  |  |  |  |  |  |  |
|  |  |  |  |  |  |  |  |  |  |
